# Spatial-Temporal Survey and Occupancy-Abundance Modeling To Predict Bacterial Community Dynamics in the Drinking Water Microbiome

**DOI:** 10.1128/mBio.01135-14

**Published:** 2014-05-27

**Authors:** Ameet J. Pinto, Joanna Schroeder, Mary Lunn, William Sloan, Lutgarde Raskin

**Affiliations:** ^a^ Infrastructure and Environment Research Division, School of Engineering, University of Glasgow, Glasgow, United Kingdom; ^b^ Department of Civil and Environmental Engineering, University of Michigan, Ann Arbor, Michigan, USA

## Abstract

Bacterial communities migrate continuously from the drinking water treatment plant through the drinking water distribution system and into our built environment. Understanding bacterial dynamics in the distribution system is critical to ensuring that safe drinking water is being supplied to customers. We present a 15-month survey of bacterial community dynamics in the drinking water system of Ann Arbor, MI. By sampling the water leaving the treatment plant and at nine points in the distribution system, we show that the bacterial community spatial dynamics of distance decay and dispersivity conform to the layout of the drinking water distribution system. However, the patterns in spatial dynamics were weaker than those for the temporal trends, which exhibited seasonal cycling correlating with temperature and source water use patterns and also demonstrated reproducibility on an annual time scale. The temporal trends were driven by two seasonal bacterial clusters consisting of multiple taxa with different networks of association within the larger drinking water bacterial community. Finally, we show that the Ann Arbor data set robustly conforms to previously described interspecific occupancy abundance models that link the relative abundance of a taxon to the frequency of its detection. Relying on these insights, we propose a predictive framework for microbial management in drinking water systems. Further, we recommend that long-term microbial observatories that collect high-resolution, spatially distributed, multiyear time series of community composition and environmental variables be established to enable the development and testing of the predictive framework.

## IMPORTANCE

Safe and regulation-compliant drinking water may contain up to millions of microorganisms per liter, representing phylogenetically diverse groups of bacteria, archaea, and eukarya that affect public health, water infrastructure, and the aesthetic quality of water. The ability to predict the dynamics of the drinking water microbiome will ensure that microbial contamination risks can be better managed. Through a spatial-temporal survey of drinking water bacterial communities, we present novel insights into their spatial and temporal community dynamics and recommend steps to link these insights in a predictive framework for microbial management of drinking water systems. Such a predictive framework will not only help to eliminate microbial risks but also help to modify existing water quality monitoring efforts and make them more resource efficient. Further, a predictive framework for microbial management will be critical if we are to fully anticipate the risks and benefits of the beneficial manipulation of the drinking water microbiome.

## INTRODUCTION

The abundant (1) and diverse (2) drinking water (DW) microbiome migrates from the DW treatment plant (DWTP) through the distribution system (DWDS) into our built environment (i.e., homes, schools, etc.) (3). Drinking water emerging from the tap may contain up to millions of microbial cells per liter, including bacteria (4), archaea (5), eukaryotes (6, 7), and viruses (8), which together constitute a complex microbial community. The DW treatment field has traditionally focused on managing the detrimental effects of these microbes on public health (2, 9), the integrity of the water infrastructure (e.g., microbially induced corrosion [10]), and the aesthetic quality of water (11). However, the DW microbiome can also be used beneficially to remove pollutants through the operation of biofiltration processes (12). To minimize detrimental microbial effects, the DW industry tries to control microbial activity by using disinfection and by limiting the availability of microbial growth substrates. It is indeed remarkable that the DW microbiome persists under extreme conditions of acute stress (primary disinfection) and chronic stress (secondary disinfection) and very low substrate concentrations. The study of this persistent microbial community in biofilms (13, 14), suspended particles (15), and bulk water (4, 16) through the use of molecular tools (17–19) has significantly improved our understanding of the DW microbiome. The cumulative knowledge generated by these and several other studies have highlighted the effects of DW infrastructure (20), disinfection strategies (8, 21), seasons (22), water age (23), and process operations (4, 24) on microbial community composition. Recently, the possibility of beneficially controlling the DW microbiome through direct process interventions (4) and infrastructure changes (25) has also been raised. However, manipulating the DW microbiome to benefit consumers necessitates the ability to confidently predict its dynamics within existing DWTPs and DWDSs.

The current study presents data from a 15-month survey of bacterial communities in a full-scale DWTP and DWDS and shows evidence that predicting the dynamics of the DW microbiome is possible. In doing so, we provide novel insights that will help the DW field test a set of hypothesis-driven strategies and develop a predictive framework for microbial management. We demonstrate that the bacterial community in the DWDS (i) clusters closely with the DWTP community while exhibiting small localized DWDS effects, (ii) exhibits spatial patterns (distance decay and dispersivity) that conform to the layout of the DWDS, (iii) displays temporal trends that indicate annual reproducibility, (iv) is driven by two seasonal clusters with distinct cluster-level network characteristics, and (v) exhibits robust interspecific occupancy-abundance relationships that utilize data from all detected taxa, linking detection frequency of taxa to their observed relative abundance. Based on these five major findings, we suggest that the collection of fine-scale spatial and temporal data through the establishment of long-term DW ecological observatories should allow the forecasting of the DW microbiome. The ability to predict the DW bacterial community has the potential to impart significant cost savings to the water industry by improving the efficiency of water quality monitoring, reduce risk to public health by helping to eliminate microbial risks before they are manifested, and also pave the way toward exploiting the multiple benefits that a functionally diverse and structurally robust microbial community offers.

## RESULTS

### *Proteobacteria* dominate the DW bacterial community.

**FIG 1:**
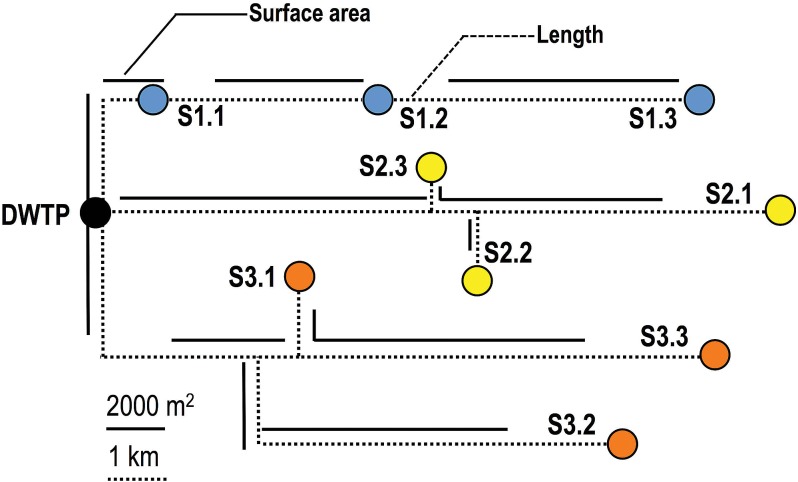
Schematic showing the sampling points at the DWTP (black circle) and in the three sectors of the DWDS (blue circles, sector 1; yellow circles, sector 2; orange circles, sector 3) included in this study and the layout of the pipe network connecting them. Dashed lines are scaled to the pipe lengths, and bold lines are scaled to the pipe surface area between any two sampling points. Scale bars for pipe length and surface area are shown at the bottom of the figure. Sampling points in sector 1 are located along a linear flow path, while sectors 2 and 3 have two and three branches, respectively.

**FIG 2:**
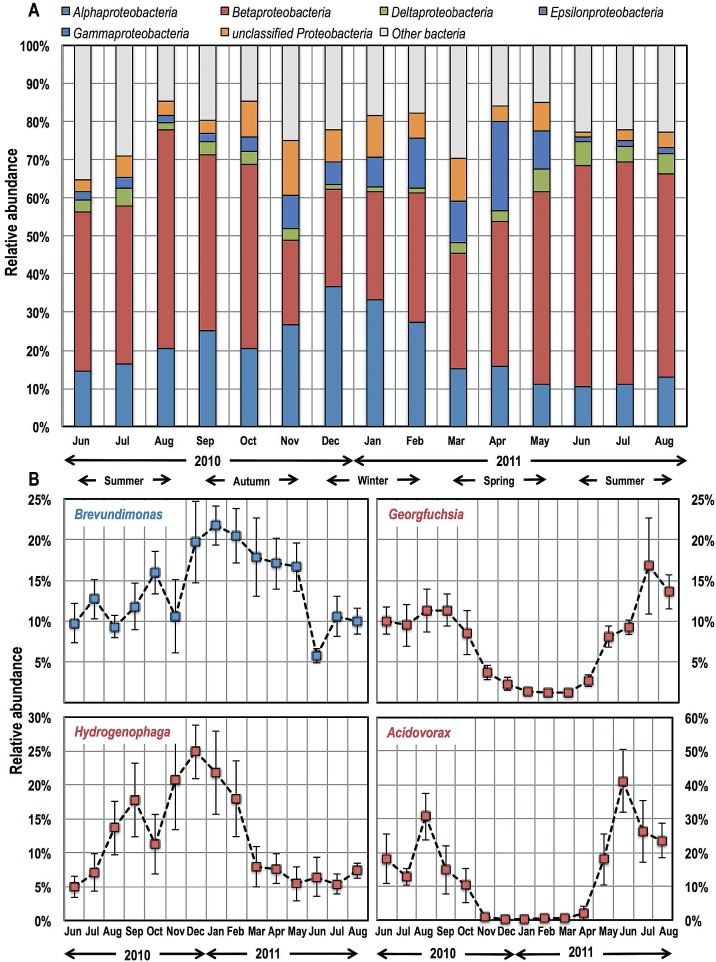
(A) Class-level relative abundances based on all sequences detected at all sampling locations within each month. Five classes of *Proteobacteria* are shown separately, while the remaining 19 phyla are shown as a single group (i.e., other bacteria). (B) The changes in relative abundance of one alphaproteobacterial OTU (blue) are compared to those of three betaproteobacterial OTUs (red) for each of the sampling months. Error bars for each data point represent standard deviations in relative abundance across all monitoring locations within each month.

Monthly water samples were collected from the clean water reservoir at the DWTP and at nine different locations in three sectors (S1, S2, and S3) of the DWDS of Ann Arbor, MI (Fig. 1), for a period of 15 months. The bacterial community in all samples was taxonomically diverse and consisted of a total of 4,369 operational taxonomic units (OTUs) at a 97% similarity cutoff. *Proteobacteria* was the dominant phylum in all samples, with *Beta*-, *Alpha*-, and *Gammaproteobacteria* constituting 42, 19, and 6.5% of the sequences, respectively (Fig. 2A). *Betaproteobacteria* dominated during the summer months (58% of all sequences in July 2011 were betaproteobacterial), while *Alphaproteobacteria* reached their maximum relative abundance in winter (37% in December 2010). This class-level proteobacterial preference for particular times of the year (summer versus winter) was not always reflected at the individual OTU level. This is particularly true for the most abundant OTUs within the data set (Fig. 2B). For example, the dominant betaproteobacterial OTU, i.e., *Hydrogenophaga* (family, *Comamonadaceae*), exhibited peak relative abundance in the colder months (December 2010), like an alphaproteobacterial OTU, *Brevundimonas* (family, *Caulobacteraceae*), which showed high relative abundance in the winter (25%). In contrast, *Acidovorax* (family, *Comamonadaceae*) and *Georgfuchsia* (family, *Rhodocyclaceae*), two dominant betaproteobacterial OTUs, exhibited peak relative abundances in the summer, which was consistent with overall taxonomic classification results. Candidate phylum OD1 was the second-most-abundant phylum, constituting 6.5% of the sequences overall and exhibiting peak relative abundance in the summer months (June 2010, 12%; June 2011, 13%), and 19 other phyla were detected at varying levels throughout the year (see Table S1 in the supplemental material).

**FIG 3:**
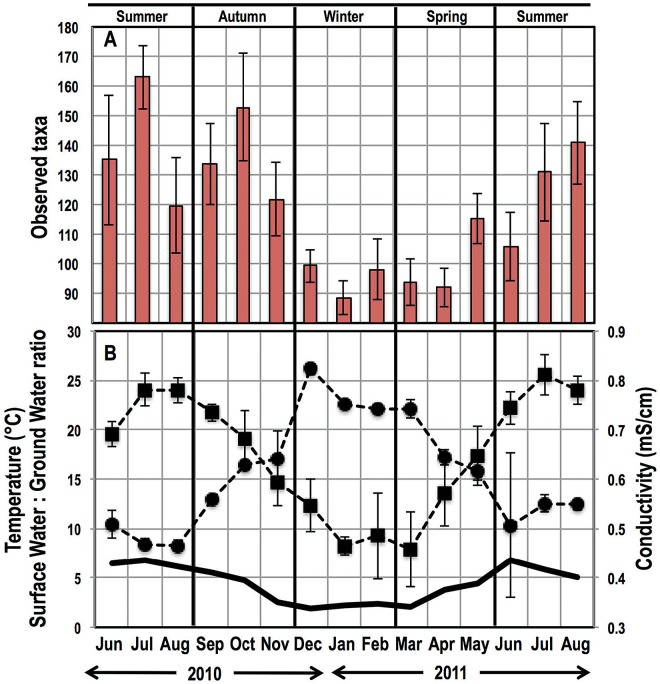
Temporal change in richness (i.e., observed OTUs) averaged across sampling locations within each month (A) correlates with water temperature (black squares), conductivity (black circles), and the surface water/ground water ratio (smooth black line) (B). Error bars indicate standard deviations in respective metrics/measurements across sampling locations within each month.

The richness (observed number of OTUs) showed a strong seasonal trend, with lower richness levels observed in the winter (December 2010 to February 2011) and spring (March 2011 to May 2011) than in the summer (June 2010/2011 to August 2010/2011) and autumn (September 2010 to November 2010) months (*P <* 0.0001) (Fig. 3A). This richness was strongly correlated with water temperature (Pearson’s *R* = 0.74, *P* < 0.05), conductivity (Pearson’s *R* = −0.70, *P* < 0.05) (Table S2), and the surface water/ground water blend ratio used as source water (Pearson’s *R* = 0.67, *P* < 0.05), which also varied as a function of season (Fig. 3B). However, we did not observe similar correlations for the structure-based alpha diversity metrics, such as Shannon evenness and nonparametric Shannon diversity (Fig. S1).

### Spatial patterns and distance decay in the distribution system.

**FIG 4:**
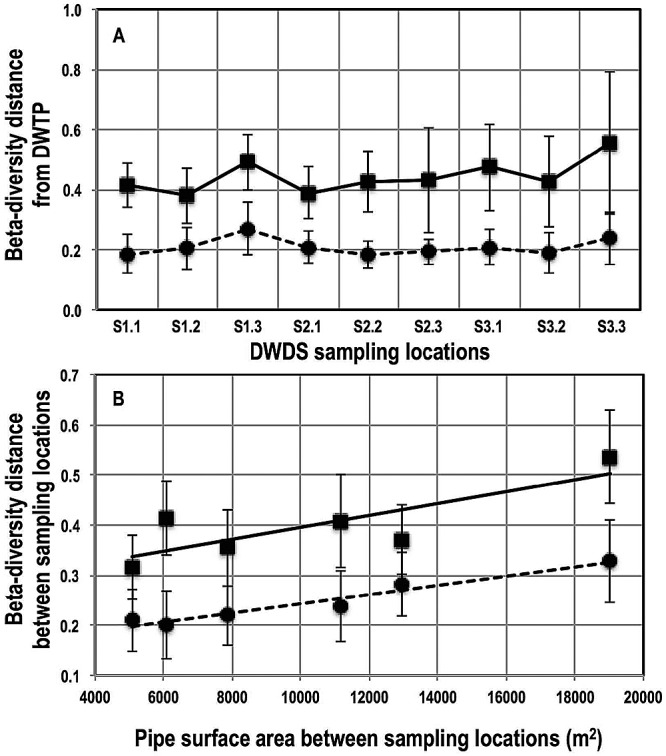
(A) Bray-Curtis distances (black squares) and weighted UniFrac distances (black circles) of the DWDS sampling locations (*x* axis) from the reservoir at the DWTP averaged over the duration of the study. (B) Correlations between Bray-Curtis distances (black squares) and weighted UniFrac distances (black circles) of bacterial communities sampled at any two locations in sector 1 and the pipe surface area connecting them. Error bars for both plots show variability over the 15-month sampling campaign in the respective beta diversity metric.

The bacterial community in all DWDS sampling locations clustered closely with the water leaving the DWTP within each sampling month. The average dissimilarity in community structures between DWDS locations and the DWTP was approximately 20 to 40% depending on the beta diversity metric used (weighted UniFrac distance, 0.23 ± 0.07; Bray-Curtis distance, 0.44 ± 0.14) (Fig. 4A). The bacterial communities at the DWTP and DWDS locations were not significantly different for any of the sectors based on the Bray-Curtis distance (sector 1, 0.42 ± 0.10; sector 2, 0.42 ± 0.12; sector 3, 0.49 ± 0.20) or weighted UniFrac distance (sector 1, 0.24 ± 0.08; sector 2, 0.22 ± 0.05; sector 3, 0.24 ± 0.08). The DWDS samples clustered more closely with the DWTP samples between the months of November 2010 and April 2011 (weighted UniFrac distance, 0.17 ± 0.03) than in the warmer months (weighted UniFrac distance, 0.24 ± 0.09). A significant number of OTUs were specific to each sampling location, and these constituted between 7 and 16% of the relative abundance of sequences and 15 to 30% of the membership over the duration of the sampling campaign (Fig. S2). However, we did not see any site-specific trends of increased or decreased richness of the bacterial community, nor did we observe any consistent trends with respect to the changes in the richness of the bacterial community between the water leaving the plant and the water emerging from the sampling locations in the DWDS.

To further assess the relationship between distance and change in a bacterial community, we related dissimilarity in community structure between two points within each DWDS sector to pipe attributes connecting them. DWDS sector 1 showed the strongest Pearson’s correlations between DWDS characteristics and differences in bacterial community structure (the 15-month average) by weighted UniFrac/Bray-Curtis distance correlations (Fig. 4B), with total length, total surface area, age-weighted length, and age-weighted surface area being 0.99 (*P =* 0.0002)/0.68 (*P =* 0.13), 0.97 (*P =* 0.0009)/0.81 (*P =* 0.048), 0.96 (*P =* 0.0022)/0.75 (*P =* 0.084), and 0.9 (*P =* 0.0145)/0.85 (*P =* 0.0317), respectively. Sectors 2 and 3 did not exhibit similar significant correlations between DWDS pipe characteristics and bacterial community structure.

We tested for the presence of localized community structure by performing analysis of similarity (ANOSIM) tests by grouping samples based on either sampling location or DWDS sector. Though differences were significant (*P <* 0.0001), ANOSIM demonstrated poor support for dissimilarities between the different sectors of the DWDS (by ANOSIM, *R* for sectors 1 and 2 [*R*_S1-S2_] = 0.19, *R*_S1-S3_ = 0.25, and *R*_S2-S3_ = 0.004). Further, the global ANOSIM results based on grouping sequences by monitoring location, though significant, were very weak (*R* = 0.1, *P <* 0.001). The highest community dissimilarities (ANOSIM, *R* ≥ 0.3) were observed between points that were most distantly located from each other across the three DWDS sectors (Fig. 1). For example, S3.2 showed nearly equal differences from locations S1.1 and S1.2 (*R* = 0.31 to 0.32) while exhibiting the greatest dissimilarity from S1.3 (*R* = 0.46) by the Bray-Curtis distance metric. Similarly, S3.3 was most different from locations in sector 1 in the increasing order S1.1 (*R* = 0.3), S1.2 (*R* = 0.33), and S1.3 (*R* = 0.35), which is consistent with the increasing pipe lengths and surface areas between these locations. This was also consistent with comparisons of sampling locations between sector 2 and sector 1.

### Comparing temporal and spatial trends in bacterial community structure.

Table S3A and S3B show summaries of permutational analyses of variance (PERMANOVA) and ANOSIM tests, respectively. Samples were compared using structure-based (Bray-Curtis, weighted UniFrac) and membership-based (Jaccard, unweighted UniFrac) metrics by grouping them based on month versus DWDS location and season versus DWDS sector. Both PERMANOVA and ANOSIM tests for the four different metrics showed that variability among samples was best explained by temporal groupings. DWDS location- and sector-based groupings had significant but very weak support (PERMANOVA, *R*^2^ ≤ 0.08; ANOSIM, *R* ≤ 0.05), compared to monthly and seasonal groupings. To further compare temporal and spatial variabilities, we tested for significant differences in beta dispersivity by grouping samples using spatial (DWDS location, sector) and temporal (month, season) criteria. Beta dispersivity relates to the scatter of all samples within a defined group (spatial or temporal) around the centroid of that particular group and provides a measure of variability in bacterial community between samples within the group. We observed no significant differences in beta dispersivity based on DWDS location and sector over the duration of the study, irrespective of the distance metric used (ANOVA, *P* > 0.05). However, we found significant support for monthly and seasonal differences in beta dispersivity of samples (all ANOVA, *P* < 0.05). The temporal patterns in beta dispersivity were further confirmed by the fact that the winter 2010/2011 samples showed significantly lower dispersivity than the other seasons (Tukey’s honestly significant difference [HSD] = −0.1 to −0.05, *P* = 0 to 0.007). Further, the two transition seasons, autumn of 2010 and spring of 2011, did not show significant differences in levels of dispersivity (Tukey’s HSD = 0.00004, *P* = 1), while summer of 2010 and 2011 also showed similar levels of beta dispersivity (Tukey’s HSD = −0.03, *P* = 0.12).

### Temporal trends in bacterial community structure and relationship to environmental parameters.

**FIG 5:**
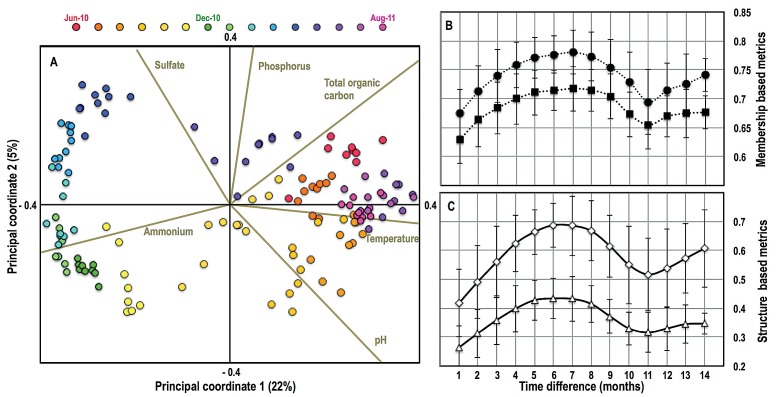
(A) Principal-coordinate biplot showing the temporal variability of the bacterial community structure constructed using the Bray-Curtis distance metric. Data points are colored by month, and the legend is provided above the plot. Environmental and process parameters, with significant Pearson’s correlation (*P* < 0.000001) with either of the two principal axes, are shown here. Beta diversity distance between samples as a function of time difference (months) between them using membership-based (Jaccard [black circles]; unweighted UniFrac [black squares]) (B) and structure-based (Bray-Curtis [open diamonds]; weighted UniFrac [open triangles]) (C) metrics. Error bars in panels B and C indicate variability in beta diversity distance for sample comparisons for each time difference.

The bacterial community also exhibited a cyclical temporal pattern, with strong evidence of clustering of bacterial community structures in the summer samples collected 1 year apart (Fig. 5A). This trend was consistent irrespective of the beta diversity metric of choice, indicating support for this annual cyclical pattern. To assess whether this cyclical temporal pattern may provide the opportunity to predict changes in community structure, we calculated pairwise time distances between samples collected at each sampling location across all months. Specifically, we calculated the pairwise beta diversity distances between two time points and the variances associated with each of these averages for all combinations of sampling points (Fig. 5B and C). The beta diversity distance between samples increased as a function of time and peaked at a 6- to 7-month time difference, followed by a drop in dissimilarity level (i.e., increased similarity) until the 11- to 12-month mark, followed by an increase.

Changes in community structure correlated very weakly when all measured environmental parameters were considered together (by Mantel’s test, *P* < 0.001; *r*_Bray-Curtis_ = 0.23, *r*_Jaccard_ = 0.23, *r*_weighted UniFrac_ = 0.23, *r*_unweighted UniFrac_ = 0.20). Rather, different environmental and process parameters explain the changes in bacterial community structure at different times of the year (Fig. 5A). For example, temperature is correlated with community structure in the summer, pH appears to be important during the transition from summer to autumn, ammonium concentrations are most relevant in late autumn and winter, sulfate and phosphate concentrations are important in spring, and total organic carbon correlates with community structure during the transition from spring to summer. These temporal relationships between community structure and water quality parameters are consistent with changes in most of the parameters that vary due to deliberate process modifications (pH, ammonium, phosphate) and those that vary due to environmental conditions (temperature, total organic carbon, sulfate) during the course of the sampling campaign (Table S2).

### Seasonal clusters within the bacterial community.

**FIG 6:**
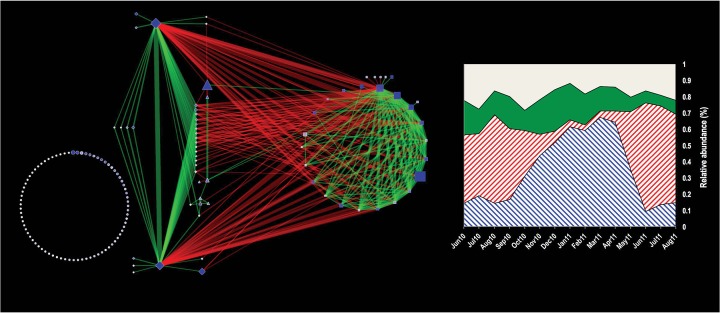
(Left) Network visualization of associations between OTUs with a detection frequency greater than 30%. Each node depicts an individual OTU, with the size of the node corresponding to its log (1 + *X*)-normalized relative abundance and the color depicting its detection frequency (white, 30%; dark blue, 100%). The edges indicate associations between OTUs; the thickness of an edge is scaled to the association strength (MIC range = 0.4 to 1), and color indicates positive (green) or negative (red) associations. Square, diamond, and triangle OTU nodes belong to clusters 1, 2, and 3, respectively, while the group of isolated taxa consists of circular OTU nodes. (Right) Relative abundances of the clusters discovered through association analyses for each sampling month. Blue hatched, cluster 1; red hatched, cluster 2; solid green, cluster 3; solid white, isolated taxa.

We measured the contribution of OTUs toward changes in the DW bacterial community by performing Mantel’s test between distance matrices constructed by including a subset of OTUs from the entire data set of 4,369 OTUs. OTUs were selected either based on their average relative abundance across all samples (range, 0.001 to 10%) or by the percentage of samples in which they were detected (range, 2 to 100%). OTUs with an overall relative abundance greater than 0.02% (number of OTUs = 245) or an overall detection frequency greater than 30% (number of OTUs = 209) demonstrated bacterial community changes (Mantel’s *r >* 0.999, *P* = 0) similar to those when all detected OTUs were considered (Fig. S3). This indicates that a small subset of OTUs (~5%) is primarily responsible for the observed spatial and temporal trends and may constitute the core bacterial community. We selected OTUs using a detection frequency threshold of 30% (Mantel’s *r* > 0.995, *P* = 0) to further identify OTUs responsible for the strong temporal trends discussed above (Fig. 3A). To do this, we subsampled the entire data set 100 times so that all samples had the same number of sequences (*n* = 834, determined by the sample with the lowest number of sequences) within each subsampling event. We estimated the detection frequency of each OTU within each subsampling event. If an OTU was detected over a frequency of 30% for all 100 subsampling events, it was selected for further analyses. This reduced the number of OTUs included in further analyses from 209 (at an overall detection frequency of 30%) to 145 (at a detection frequency for each subsampling event of 30%). Next, we performed association analyses using maximal information-based nonparametric exploration (MINE) (26) for these 145 OTUs within each subsampling event. MINE analyses generate a maximum information coefficient (MIC) to quantify the strength of association between any two OTUs by considering a suite of relationship types. To ensure that the associations being detected were robust, we took additional stringency measures. First, we performed MINE analyses on each of the 100 subsampling events with the condition that MICs were calculated between two OTUs only if they cooccurred in at least 70% of the samples to ensure that each association was supported by a minimum of 21 data points. Second, we considered an association valid only if it was significant in at least 10 of the 100 subsampling events (MIC > 0.4, Bonferroni-corrected *P* < 0.05) and the sign of the slope of the linear regression (i.e., positive or negative) was consistent every time it was discovered. We found a total of 283 significant OTU-OTU associations for 66 of the 145 OTUs (168 positive, 115 negative associations), while 79 OTUs did not show any significant associations and appeared to be isolated (Table S4). This resulted in a sparsely associated community with an overall clustering coefficient of 0.288 (i.e., ~29% of all possible OTU-OTU associations were satisfied), estimated using the Network Analyzer option in Cytoscape (version 3.0.1) (27). The association of these OTUs is shown in the network plot (Fig. 6). The number of associations of any particular OTU within the network did not correlate with either its relative abundance or the frequency with which it was detected.

The 66 connected OTUs were separated into three distinct clusters based on the types of associations between them (positive or negative associations [see below]). These clusters were designated clusters 1, 2, and 3 and consisted of 24, 32, and 7 OTUs, respectively, with three OTUs not belonging to any particular cluster. The two large clusters (cluster 1 and cluster 2) were characterized by OTUs exhibiting only positive associations within their respective clusters and negative associations across clusters, indicating that they represented clusters within the larger communities with distinct temporal preferences that coexisted within the DW system (Fig. S4) but dominated the bacterial communities at different time points. The third cluster exhibited positive and negative associations with both large clusters, although the numbers of associations were limited. Cluster 1 was dominated by a betaproteobacterial OTU that was classified in the genus *Hydrogenophaga* (relative abundance, 13.9% ± 4.7%), a gammaproteobacterial OTU that was classified in the genus *Pseudomonas* (relative abundance, 4.4% ± 6.2%), and two other proteobacterial OTUs that could not be classified to the genus level (relative abundances, 3.9% ± 3.2% and 2.2% ± 2.1%). Cluster 2 was dominated by betaproteobacterial OTUs that were classified to the genus *Acidovorax* (relative abundance, 13.3% ± 13%) and *Georgfuchsia* (relative abundance, 7.4% ± 5%) and an OTU that was classified to the phylum OD1 (relative abundance, 3.9% ± 2.4%). Further analyses using the total relative abundances of the OTUs within the three clusters highlighted distinct temporal patterns (Fig. 6, right). Specifically, cluster 1 was the dominant cluster from October 2010 through April 2011, cluster 2 was dominant from June 2010 to September 2010 and June 2011 to August 2011, while cluster 3 demonstrated stable relative abundance throughout the duration of the sampling campaign. This allowed us to categorize cluster 1 as the winter cluster and cluster 2 as the summer cluster. The two major clusters showed significant phylogenetic differences (unweighted UniFrac score, 0.7877, *P* < 0.01), with cluster 2 being dominated by *Beta*- and *Deltaproteobacteria*, while cluster 1 was composed to a large extent of *Alpha*- and *Gammaproteobacteria*. The two clusters also showed different within-cluster network characteristics. Specifically, cluster 2 exhibited nearly 4-fold-higher network density (0.38) than cluster 1 (0.1). The two dominant OTUs in cluster 2 shared positive and equally strong associations with a majority of the other medium- to low-abundance satellite OTUs within this cluster. Interestingly, these medium- to low-abundance OTUs did not exhibit any significant associations with each other in cluster 2. In contrast, cluster 1, while clearly exhibiting the presence of a hub OTU based on the number of associations (genus, *Pseudomonas*), also had significantly more inter-OTU associations than cluster 2.

### Proportionality between frequency of detection and relative abundance of OTUs.

**FIG 7:**
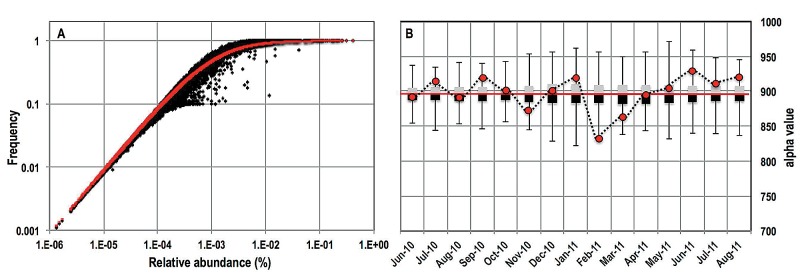
(A) The Hanski-Gyllenberg model (Table 1) resulted in the best fit (red line) to the frequency-abundance data from the Ann Arbor DW system. The OTU abundances were estimated by averaging the relative abundances of each taxon across all sampling locations within each sampling time point (i.e., month); frequency represents the proportion of samples in which the OTU was detected within each sampling time point. (B) The fitted value of α for each month (red circles) was within bounds of the permuted random α values (box plot: black, first quartile; gray, third quartile; whiskers, minimum and maximum values) obtained from 1,000 permutations and the overall α value for the entire data set (red line). February 2011 was the exception, with a lower-than-expected α value.

The 4,369 OTUs showed strong proportionality between their average relative abundance (μ) and the frequency (*f*) with which they were detected (number of samples in which they were detected). To establish a quantitative relationship between the μ and *f*, we expressed the relationship between these two variables in the form of established interspecific occupancy abundance models (IOAMs) (28). Traditionally, occupancy refers to the number of patches or sites on a two-dimensional fixed landscape where species, often plants, are present in samples. However, these IOAMs have also been applied to motile fauna, for example, birds (29) or fish, which are not fixed in space but will pass through landscape patches. Our application of IOAMs is a reasonable extension to planktonic communities that move through a fixed location in space in a DWDS. We estimated the μ and *f* for each OTU within each of the 15 time points (i.e., months) after subsampling the data set 100 times and then averaged the μ and *f* across these 100 subsampling events, which resulted in a maximum of 15 nonzero data points per OTU. Using these data, we tested a number of IOAMs (28) with the aim of using a minimal number of parameters to obtain a best-fit model. The five tested models and their goodness of fit are shown in Table 1. The Poisson model (30) did not converge to a solution, while it was calibrated to the Ann Arbor data set, while the negative binomial (31) and the power (32) models converged to similar nonoptimal solutions. The two models that showed the best possible fit to our data set were the Nachman (33) and Hanski-Gyllenberg (34) models. For the Ann Arbor data set, the Hanski-Gyllenberg model provided the best fit, with an α value of 896 (Fig. 7A). To determine if the estimated α showed temporal variability, we fitted the Hanski-Gyllenberg model to frequency-abundance plots for each month and obtained their respective α values. Then, we randomized the month assignments for each OTU while maintaining their sampling location assignment and refitted the Hanski-Gyllenberg model to estimate the randomized α value for each month. This permutational exercise was repeated 1,000 times to determine the range of random α values for each month, and these were compared to the α values obtained for the actual data (Fig. 7B). Figure 7B shows that values of the overall fitting parameter calculated for the entire data set and that for each month (sparing February 2011) were within the bounds of those estimated by permutation tests.

**TABLE 1:** Interspecific occupancy-abundance modeling with the maximum-likelihood-based determination of the best-fit model^*a*^

Model	Model form	Maximum likelihood	Mean absolute deviance	Reference
Poisson	$P=1-e^{-\mu }$	−109082.22	0.1716	30
Nachman	$P=1-e^{-\alpha \mu^{\beta }}$	−10659.83	0.0359	33
Hanski-Gyllenberg	$P=\frac{\alpha \mu^{\beta }}{1+\alpha \mu^{\beta }}$	−10622.13	0.0347	34
Power	$P=\alpha \mu^{\beta }$	−109022.28	0.1716	32
Negative binomial	$P=1-{\left(1+\frac{\mu }{k}\right)}^{-k}$	−109021.05	0.1716	31

^a^ For the model-fitting exercise, β was fixed as 1. Thus, the model fitting involved only iterative variation in α values, followed by estimation of maximum likelihood and mean absolute deviance.

## DISCUSSION

### A small subset of bacteria dominates the DWDS bulk water community, despite its high diversity.

We detected 4,369 OTUs across 20 different phyla in 138 water samples collected over a period of 15 months from the clean water reservoir and at nine different locations in the DWDS of Ann Arbor, MI. It is important to note that we did not discriminate between the detected OTUs or adjust their relative abundances to reflect their viability status. The aforementioned OTUs were detected after PCR amplification and sequencing from total DNA extracts. Thus, it is likely that some of the sequences used in this study may originate from nonviable or dead cells. There are several methods to discriminate between live and dead cells depending on the viability metric of choice (e.g., membrane damage [35, 36], RNA [37], enzymatic activity [38], and DNA damage [39]). However, none of these approaches, alone or in combination, have thus far demonstrated robust discrimination between live and dead cells. Our approach of PCR amplification and sequencing from total extracted DNA represents a conservative view of the bacterial community under investigation, and we recommend that future studies should attempt to test our findings using one or a combination of the mentioned viability screening approaches.

Consistently with previous findings, *Proteobacteria* was the most dominant phylum (19, 22, 40–42) and constituted in excess of 60 to 70% of the bacterial community for any given sample. Further, among the proteobacteria, *Alphaproteobacteria* were most abundant during the winter months, whereas *Betaproteobacteria* were dominant during the summer months (22). However, this seasonal dynamic was not limited to *Proteobacteria*. Sequences from candidate phylum OD1 were also quite abundant and reached their highest relative abundances in the same month, 1 year apart (i.e., 12% in June 2010 and 13% in June 2011), while showing low abundance in the winter. Similarly, *Acidobacteria* (relative abundance, 0.6% ± 0.7%) and *Gemmatimonadetes* (relative abundance, 0.17% ± 0.2%) showed higher relative abundances in the warmer periods of the year (summer and autumn), while *Chlorobi* (relative abundance, 0.03% ± 0.03%) showed a preference for the winter. Despite the diversity of OTUs and broad taxonomic diversity, only ~5% of the detected OTUs were required to explain the changes in community structure across 138 samples based on Mantel’s test (Fig. S3). This finding is not unusual, and a similar explanatory power of a small subset of OTUs has also been demonstrated in other unrelated aquatic systems (43). This is not to say that the rest of the OTUs are irrelevant, as even a low pathogen presence can render DW unsafe for public consumption. Rather, the explanatory power of the subset of OTUs may allow for the development of targeted monitoring of select OTUs as a means of quickly assessing the changes in the overall bacterial community structure.

### Differences in bacterial community structures conform to the DWDS layout.

DWDSs consist of a complex underground network of pipes and fittings of different materials, ages, and sizes that are connected to each other to transport treated water from the DWTP to our built environment. The bulk water bacterial community may change in the DWDS due to regrowth resulting from changes in substrate availability or disinfectant residual concentration (44), exchange of biomass with the biofilm growing on the pipe surfaces (13), and numerous ecologically relevant microbe-microbe interactions (2) (e.g., competition for resources in an oligotrophic environment [45] and bacterium-amoeba interactions [46]). Quantifying these changes is further complicated by the highly heterogeneous nature of the DWDS network in terms of layout, composition, and the actual water path (and associated pipe biofilm exposure), which varies as a function of localized water demand. The sampling locations included in this study were connected to the DWTP (shortest water path) by 881 pipe sections made up of five different materials; in total, they were approximately 46 km in length and had 72,088 m^2^ of pipe surface area and an average age of 40 ± 23 years (range, 91 years to 2 months) between them (Table S5). All of these parameters introduce variability in how the DWDS characteristics influence the bacterial community as it migrates from the DWTP to each DWDS sampling location. Given these compounding parameters and the complexity of the DWDS layout, it may be impossible to separate out all individual mechanisms responsible for changes in bacterial community structure in a full-scale DWDS.

Despite these confounding complexities, we have made several key findings that provide useful insight into how the DWDS spatial structure affects the bacterial community in the bulk water. First, approximately 15 to 30% of the OTUs in water collected from each DWDS sampling location were specific to their location, and they constituted between 7 and 16% of the overall relative abundance. These OTUs are most likely introduced into the bulk water due to detachment of biofilms in the neighborhood of each sampling location or possibly even microbial ingress into the DWDS. This is in contrast to recent findings that found little to no overlap between bulk water and biofilm communities (13). However, such observations may arise due to the difficult task of collecting representative pipe biofilm samples, which can be extremely spatially heterogeneous (e.g., due to effects of pipe materials [47]). The cumulative effect of localized seeding of the bulk water by the pipe biofilm is further highlighted by the fact that only the most distant points in different sectors of the DWDS exhibited significantly different community structures. It is unlikely that such changes are purely due to dynamics within bulk water (i.e., regrowth), since some of these distant points were approximately equal in distance from the DWTP and hence the bulk water communities had traveled similar distances before emerging from the DWDS locations. For example, S3.2 in sector 3 exhibited maximum dissimilarity to S1.3 (ANOSIM *R* = 0.46) in sector 1, while the pipe length and surface area connecting these two points to the DWTP was between 11 and 13 km and 19,000 and 19,700, respectively. The differences in bacterial community structure between these points most likely arose from cumulative influences of pipe biofilms, which may be different for each sector, on the bulk water community.

We also show that the change in bacterial community structure in the DWDS can be quantified and that accurate estimates of distance decay (i.e., decreasing similarity in communities with increasing distances between them) are possible. Specifically, for sector 1, we detected strong correlation between pipe characteristics (length, surface area, pipe age) and changes in community structure (Pearson’s *R* = 0.81 to 0.99), irrespective of the beta diversity metric of choice. This is a significant finding which may allow us to predict how much a bacterial community changes after leaving the DWTP as it moves through the DWDS. Though we did not see similarly strong correlations for the other two sectors of the DWDS, this may be attributed to the differences in complexity of the network configurations for the three DWDS sectors sampled in this study. Specifically, sectors 2 and 3 had two and three branches, and thus a majority of the sampling points in these sectors had pipe sections unique to them. As a result, the biofilm effect on the bulk water community may be different for each unique section. In contrast, sector 1 had no branches and its three sampling locations presented a linearly connected water path (Fig. 1), thus allowing for a cumulative pipe biofilm effect resulting in the emergence of a robust relationship between distance and decay. We recommend that future efforts at estimating distance decay in the DWDS would be better served by selecting sampling locations that are linearly connected within subsectors of the DWDS.

### Two distinct bacterial clusters drive strong temporal trends.

Despite these localized effects, it is evident that the spatial trends in DW bacterial community were small compared to the temporal effects. In fact, for almost all sampling time points, the DWDS sampling locations clustered very closely with the water leaving the DWTP (60 to 80% structural similarity depending on the beta diversity metric used) (Fig. 4). PERMANOVA and ANOSIM also demonstrated that month and season were much stronger explanatory factors for changes in bacterial community structure than either DWDS location or DWDS sector (Table S3), irrespective of the beta diversity metric of choice. This is likely not because spatial effects are small but rather because temporal changes in the bulk water community are much stronger (Fig. 5). Such temporal changes in bacterial community structure, particularly seasonal, have also been reported previously (16, 22). However, comparison of spatial and temporal changes using OTU-level resolution in a large data set has thus far been lacking. Though it is unlikely that DWDS spatial effects will outweigh annual temporal trends (<30% location-specific membership per DWDS sampling location over 15 months), it should be possible to gain much finer insights into the effects of DWDS structure by adopting a scale-appropriate sampling strategy. Specifically, generating large amounts of data from spatially distributed samples within relevant ranges of water age should prevent temporal effects from masking spatial effects.

The temporal trends presented in this study provide important novel insights into the dynamics of the bacterial community in the Ann Arbor DW system. First, we report a cyclical pattern of the bacterial community structures (Fig. 5) in DW systems, which was supported by all beta diversity metrics, phylogeny versus OTU based and membership versus structure based. Specifically, we see significant clustering of bacterial communities two summers apart (summers of 2010 and 2011). Though such patterns have been reported in other engineered (48) and natural (49) aquatic environments, to our knowledge, this is a first report of cyclical dynamics in DW systems. The seasonal pattern exhibited by the bacterial community may arise for multiple reasons. Specifically, it may be due to the variations in blend ratios of the two source waters (i.e., surface and ground water), with a higher surface water/ground water ratio in the summer (Fig. 2). Changes in blend ratio influence the types of bacterial populations being introduced into the DWTP and DWDS as a function of season. This effect may also be attributed to process changes that are undertaken at the Ann Arbor plant on a seasonal basis. Specifically, the pH of the treated DW leaving the DWTP ranges from 9.3 in the summer to 8.9 in the winter. Subtle pH changes in combination with variations in substrate composition (source water effect) may support the survival/dominance of different bacterial populations on a seasonal basis. A third plausible reason may be that a large majority of the detected bacteria already coexist in the DW system (e.g., biofilms on the filters present in the DWTP [4]) and that a combination of process (e.g., pH) and environment (e.g., substrate composition/availability and temperature) influences their relative abundances on a seasonal basis.

Second, by tracking temporal dynamics, we have identified specific seasonal clusters for the Ann Arbor DW system. The observed temporal trends were due largely to the shift in bacterial communities from cluster 2 (summer cluster) to cluster 1 (winter cluster) and then back to cluster 2 (Fig. 6, right). Cluster 2 is dominated by two OTUs with a large number of low-abundance satellite OTUs, which do not associate with each other but only with the two dominant OTUs, resulting in a poorly associated network (network density = 0.1). In comparison, cluster 1 has fewer but well-connected OTUs with a significantly higher network density (0.38). The exact mechanism for differences in network densities for these clusters is worthy of future investigation, as differences in substrate availability, type of substrates, and/or temperature may play an important role.

Third, the differences in the network densities of these clusters may also explain the dispersion in bacterial community structure for each season. Specifically, a well-connected and dominant cluster 1 may be responsible for the low dispersion seen between data points in the winter rather than the sparsely connected cluster 2, which is dominant in the summer. Similarly, the transition between these two clusters during the autumn and spring months likely explains the large observed spatial dispersion in these months. The ability to identify these important clusters and correlate their abundances, network densities, and within-time-point dispersivities could be an important tool in future efforts to predict the dynamics of the DW microbiome.

### Conformity to the occupancy-abundance model indicates that distribution of OTUs is dispersal limited.

The DWDS not only selects for specific OTUs but also plays a role in affecting their dispersal within the DW system. For our data set, we found poor support for the Poisson model. The Poisson model would fit if communities are drawn at random from a single underlying taxon abundance distribution. Indeed, this model has been shown to emerge from birth-death-immigration for community composition when every change in composition arises from immigration, and hence, there are no local births. Our inability to explain the frequency-abundance relationship with the Poisson model suggests that the planktonic bacterial community is undergoing local births and deaths as it moves through the network and is dispersal limited. We do find strong support for two other versions of prevalent IOAMs. For example, the Nachman and Hanski-Gyllenberg models provided excellent fits to the Ann Arbor data set. Though both models allow for two fitting parameters, we used only one parameter (i.e., α) by fixing β to 1, thus further reducing the complexity of these simple models. By using permutation tests, we have shown that the fitted parameter does not show temporal variability, which further indicates that the IAOM fit may be a characteristic of the DW system; the best-fit model and the fitting parameter may vary from system to system. However, the consistency of the model over time suggests that deviation from the Poisson model is systematic and may reflect underlying biological or physical mechanisms. While researchers have attempted to attribute specific biological mechanisms to various IOAMs, it is widely accepted that the models are phenomenological (50). Hence, like other researchers, we can only speculate on the importance of (i) selective pressures imparted to OTUs due to process or environmental conditions (e.g., disinfectant stress, substrate availability), (ii) a balance between the regrowth of microbial cells within bulk water and the introduction of cells into the bulk water due to biofilm detachment, and (iii) the dispersal limitation of a taxon within any given DW system. For DWDS, it is a realistic prospect that careful experimentation will allow a mechanistic understanding of the phenomena captured by IOAMs. The inability to distinguish the cause of the relationship does not necessarily jeopardize its potential in predicting the frequency with which taxa will be observed in the system. We are unable to determine how the best IOAMs or model fits may vary between DW systems, as we have included only one system in our study and similar long-term data sets are currently unavailable for conducting robust comparisons. It would be a worthy future exercise to expand such an effort to multiple DW systems to understand the role and relevance of IOAMs in the management of the DW microbiome. Such efforts may provide valuable insights into the dynamics of DW systems, particularly if used in conjunction with rich spatial-temporal data sets. For example, we have fitted the models to all OTUs, thus utilizing a form of interspecific occupancy abundance. However, intraspecific occupancy abundance is also possible, such that a model is fitted for each individual OTU (51). These models could be used to estimate the rate of change of relative OTU abundances and provide insight into how rapidly an OTU may become prevalent in (invasiveness) or become eliminated from (extinction) the DWDS with increases or decreases in its relative abundance, respectively. The Ann Arbor data set presented here is not rich enough to develop this intraspecific perspective, largely because most of the OTUs do not cover the spectrum of relative abundance and frequency over the time scale of this study. For the few OTUs that do, any such intraspecific model-fitting efforts would be informed by the limited number of time points (15 months) and thus likely would not be robust.

### Path forward for a predictive microbial-management framework in DW systems.

Based on the findings in this study, we recommend five steps that will facilitate the development and testing of a predictive framework for microbial management in DW systems. First, we recommend that rather than conducting scattered sampling and analyses over a wide range of DW systems, gathering rich spatial-temporal data for select systems to first validate a framework for linking temporal, spatial, and occupancy-abundance features that can then be tested across multiple systems may be a better use of resources. The selection of these representative systems should capture a range of (i) water treatment processes, (ii) source water types and use patterns, and (iii) distribution system sizes and ages. Second, long-term multiyear temporal studies should focus on water leaving the DWTP to identify temporally relevant OTU clusters and the OTU-OTU association within and across these major clusters. Third, targeted spatial surveys within the DWDS (informed by pipe layout) should be conducted at select time points (informed by changes in environmental parameters) to measure distance-decay relationships and characterize dispersion of the bacterial community at DWDS locations around the one measured at the DWTP. This should involve collection of large amounts of spatially distributed samples within relevant water ages to decouple temporal from spatial effects. Fourth, targeted spatial surveys should be designed to calibrate IOAMs to be used in conjunction with the temporal data fitted to both the whole community and the dominant OTUs (i.e., hubs within each cluster) identified within the temporal clusters and possibly for specific taxa of interest (e.g., pathogens). Finally, OTU-OTU association data can be used to estimate the abundance of low- to medium-abundance OTUs based on the abundance of dominant hub OTUs within their respective clusters. This information will help with identification of a range of bacterial community constructs and selection of community constructs that comply with previously estimated temporal and spatial trends. By combining these five steps, a DW utility may be able to reconstruct bacterial community structure for the DWDS based on measurements at the DWTP and even predict the DWTP/DWDS community structure at operationally relevant time points in the future. Such a predictive framework for microbial management in a DW system promises multiple key benefits. First, it will enable us to predict the risk of microbial-contamination events and inform strategies to eliminate this risk before such events are manifested. Second, it will help shift the focus from water quality compliance (99.9% compliance also means 99.9% of resources are spent collecting and analyzing regulation-compliant DW samples) toward model-informed targeted sampling efforts in high-risk areas of the DWDS and centralized risk management at the DWTP. Finally, a framework that is able to predict bacterial dynamics within the existing DWTP/DWDS system will also enable us to estimate the risks and benefits of the recently suggested beneficial manipulation of the DW microbiome (4, 25) through engineering strategies that are yet to be devised and/or tested.

## MATERIALS AND METHODS

### Sampling and data collection.

Sampling was conducted in the DWTP and DWDS of Ann Arbor, MI, from June 2010 to August 2011 on a monthly basis. The treatment processes used by the Ann Arbor DWTP have been described previously (4). Briefly, the Ann Arbor DWTP treats a combination of surface and ground water with lime-softening compounds, coagulation-flocculation-sedimentation, ozonation, and dual-medium (granular activated carbon and sand) filtration, followed by addition of free chlorine and ammonia to produce chloramine prior to distribution. The average water ages in the distribution system vary between 2 and 7 days. Water samples were collected from the reservoir at the DWTP immediately before the treated water was pumped into the DWDS and at nine different sampling locations in three sectors of Ann Arbor (three locations for each sector)—central (S1), southwest (S2), and north (S3)—on three consecutive days on a monthly basis (Fig. 1). All relevant monitoring data are provided in the supplemental material (Table S2). Sample collection for chemical and bacterial analyses and DNA extraction were conducted as described previously (4), with a few differences. First, the V4/V5 hypervariable region of the 16S rRNA gene was amplified using Bact-563F (http://pyro.cme.msu.edu) and Bact-909R (52) with thermocycling conditions as described previously (4). PCR amplicons from all samples were pooled in equal mass amounts and sent to the University of Illinois DNA sequencing center, Champaign, IL, for sequencing on the 454 GS-FLX platform. In addition to performing microbial and water chemistry analyses, we obtained an inventory of the length, diameter, material, and installation year of all DWDS pipe sections connecting all DWDS sampling locations to the DWTP (Fig. 1 and Table S5) from the city of Ann Arbor.

### Sequence data processing.

PCR products for this study were twice sequenced in conjunction with samples from a different project on four-quarter regions of a plate (a total of two plates) using the 454 GS-FLX platform, yielding 939,576 sequences, with approximately 70% of the sequences belonging to this study. All sequence processing and data analyses were conducted using mothur (version 1.31.1) (53), unless indicated otherwise. The sequences were then quality filtered by specifying an average sequence quality score of 30 over a window size of 50 and removing all sequences with greater than eight homopolymers and any ambiguities. Following this, the sequences were sorted into their respective samples by allowing a maximum 2-bp mismatch with the primer and no mismatches with the multiplexing bar codes. The sequences were aligned to the SILVA seed database (54) provided through mothur (53), filtered using the vertical=T, trump=. options to ensure that all sequences were aligned along similar sections of the V4/V5 region of the 16S rRNA gene. The aligned and filtered sequences were then processed using a single-linkage algorithm (55) with a 2-bp similarity threshold; chimeras were detected using UCHIME (56), and all chimeric sequences were removed. This resulted in the retention of 420,891 sequences originating from 147 of the 150 samples used in this study, with an average of 2,864 ± 2,578 sequences per sample. A minimum sequencing depth of 834 was established, and nine samples with sequences less than this threshold were discarded, resulting in data from 138 samples. The remaining quality filtered and chimera-free sequences were classified using the RDP training set (57) available through mothur, with a threshold confidence level of 80%. Any sequences with unknown domain level taxonomy were further discarded from analyses. This extensive quality control resulted in 418,525 sequences from 138 samples, with an average of 3,032 ± 2,566 sequences per sample and with minimum and maximum numbers of sequences per sample being 834 and 16,817, respectively. Quality filtered and chimera-free sequences are publicly available through figshare (http://dx.doi.org/10.6084/m9.figshare.936611). Sequences were clustered using the average neighbor algorithm into operational taxonomic units (OTUs) using a similarity cutoff of 97%, which resulted in identification of 4,369 OTUs. The taxonomic affiliation of OTUs was assigned by obtaining a representative sequence from each OTU (otu.rep command in mothur) and then by classifying it using the RDP database at a confidence threshold of 80%.

### Sample diversity comparisons and statistics.

Alpha and beta diversity analyses were conducted to compare the samples based either on OTU-level assignment or phylogenetic placement. Specifically, OTU-based alpha diversity metrics included observed taxa (richness), nonparametric Shannon diversity, and Shannon evenness. OTU-based beta diversity metrics included Bray-Curtis distance based on similarity in community structure and Jaccard distance based on overlap in community membership. We also calculated weighted and unweighted UniFrac metrics (58) by placing all sequences on a phylogenetic tree using the clear-cut command (59) available in mothur. All aforementioned metrics were calculated after 1,000 subsamplings of the entire data set to the sample with the least number of sequences (*n* = 834) to ensure that all samples were compared at the same sequencing depth. The average beta diversity matrices for all four distance metrics, environmental data, and metadata file describing sampling location, DWDS sector, season, and month were then imported into R (http://www.r-project.org). The vegan package (60) was used to perform PERMANOVA (adonis function), which was followed by estimation of Tukey’s honestly significant difference (HSD), an ANOSIM test, beta dispersivity analyses (betadisper function), and Mantel’s test for correlating the environmental data matrix to bacterial community structure, as discussed in Results. The filter.shared command in mothur was used to select OTUs using various thresholds of relative abundance and frequency, and the subsequent Mantel’s test (see Results) was also performed in mothur. Principal-coordinate analyses using all four beta diversity metrics and data for respective biplots were generated using the pcoa and corr.axes commands in mothur.

### Association and network analyses.

We evaluated associations between OTUs using maximal information-based nonparametric exploration (MINE) (26). This metric allows for association between variables by ensuring generality (i.e., without being limited to function type) and equitability (i.e., ensuring that association strengths and significance are not affected by function type). Stringency measures to ensure robustness of detected MICs are outlined in Results. The detected MIC associations were then visualized using Cytoscape version 3.0.1 (27), and appropriate network properties were calculated using the network analyzer function in Cytoscape as discussed in Results.

### Frequency-abundance modeling.

We modeled the relationship between the average relative abundance (μ) of OTUs across the spatial-temporal sampling with the frequency (*f*) with which it was detected. We utilized the five forms of interspecific occupancy-abundance models discussed by Holt et al. (28) and fitted them using maximum likelihood. The likelihood and parameter definitions were described previously (28). To simplify the model and establish direct proportionality between relative abundance and detection frequency, we fixed the value of β to 1 and iteratively varied α so as to maximize the log-likelihood function by using the optimize function in R (version 3.0.0). We fitted all the above-described models to the frequency abundance of the entire data set after multiple subsamplings as described in Results and assessed goodness of fit using the sum of absolute differences between the observed proportions of occurrence and the fitted values. Residuals were computed as observed frequency minus the fitted frequency expected under the model (with the estimated maximum-likelihood parameter).

## SUPPLEMENTAL MATERIAL

Figure S1 Shannon evenness (A) and nonparametric Shannon diversity (B) values averaged across all sampling locations within each month did not show significant correlations to measured water quality parameters. Download Figure S1, PDF file, 0.3 MB

Figure S2 Contribution of site-specific (red) and non-site-specific (blue) OTUs to the relative abundance (top panel) and membership (bottom panel) at each sampling location for the entire sampling period. Download Figure S2, PDF file, 0.2 MB

Figure S3 Effect of OTU filtering based on detection frequency (top panel) and relative abundance threshold (bottom panel) on Mantel’s *r*. The lower the Mantel *r*, the lower the correlation between the full data set with all OTUs and the data set with a subset of OTUs based on the detection frequency or relative abundance threshold. Download Figure S3, PDF file, 0.2 MB

Figure S4 MIC associations between the three connected clusters are shown here. Isolated OTUs have been removed. Green edges indicate positive MIC associations (A), while red edges indicate negative MIC associations (B). These network visualizations are clearly demonstrated within cluster positive associations and across cluster negative associations for cluster 1 (far right) and cluster 2 (far left). Download Figure S4, PDF file, 2.2 MB

Table S1 Phylum-level classification of sequences detected at all sampling locations within each month shown as relative abundance (%). The phylum *Proteobacteria* is divided into its respective classes. Table S1, DOCX file, 0.1 MB.

Table S2 Water quality data for all sampling locations and time points. Table S2, DOCX file, 0.2 MB.

Table S3 (A) Summary of PERMANOVA results indicating the explanatory power of temporal and seasonal factors with respect to bacterial community structure. The top table compares the month and location, while the bottom table compares the season and sector for all four beta diversity metrics. (B) Global ANOSIM summaries and respective significance values for comparing samples grouped by month, location, season, and sector. Table S3, DOCX file, 0.1 MB.

Table S4 MIC statistics used to construct the network visualization (A) and taxonomy (B) of OTUs selected for this purpose. Table S4, DOCX file, 0.2 MB.

Table S5 Pipe characteristics for the three sectors of the Ann Arbor DWDS included in this study (Fig. 1). NA, not applicable. Table S5, DOCX file, 0.1 MB.
