# Supplementary material for: Spatial-Temporal Survey and Occupancy-Abundance Modeling To Predict Bacterial Community Dynamics in the Drinking Water Microbiome
Source: mBio. 2014 May 27;5(3):e01135-14. doi: 10.1128/mBio.01135-14 (PMC4045074; doi:10.1128/mBio.01135-14)

Supplementary Figure S1. (A) Shannon evenness and (B) Non-parametric Shannon diversity averaged across all sampling locations within each month did not show significant correlations to measured water quality parameters.

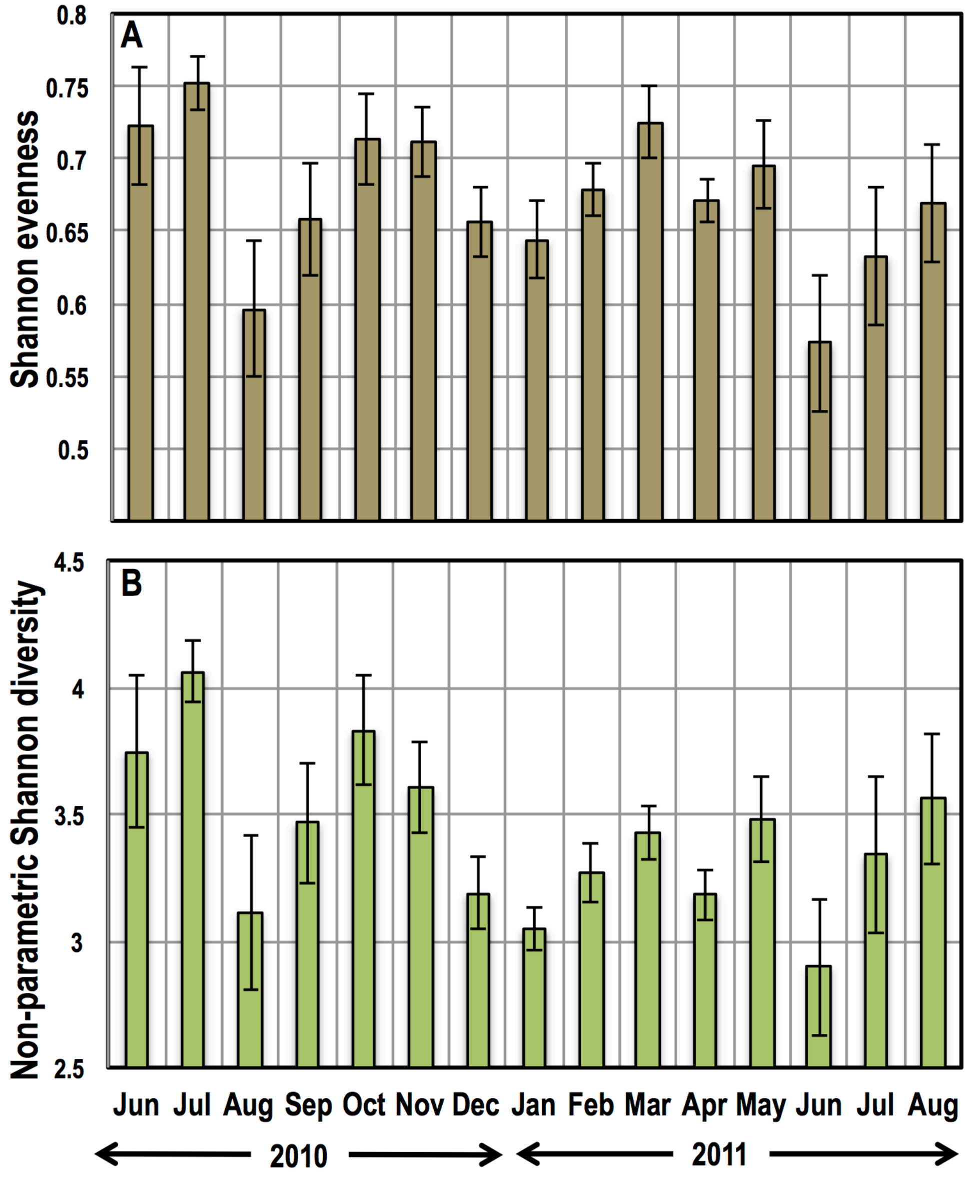

Supplement: Figure S1 — Shannon evenness (A) and nonparametric Shannon diversity (B) values averaged across all sampling locations within each month did not show significant correlations to measured water quality parameters. Download [file mbo003141850sf01.pdf]
