# Supplementary material for: Spatial-Temporal Survey and Occupancy-Abundance Modeling To Predict Bacterial Community Dynamics in the Drinking Water Microbiome
Source: mBio. 2014 May 27;5(3):e01135-14. doi: 10.1128/mBio.01135-14 (PMC4045074; doi:10.1128/mBio.01135-14)

Supplementary Figure S2: The contribution of site specific (red) and site non-specific (blue) OTUs to the relative abundance (top panel) and membership (bottom panel) at each sampling location for the entire sampling period.

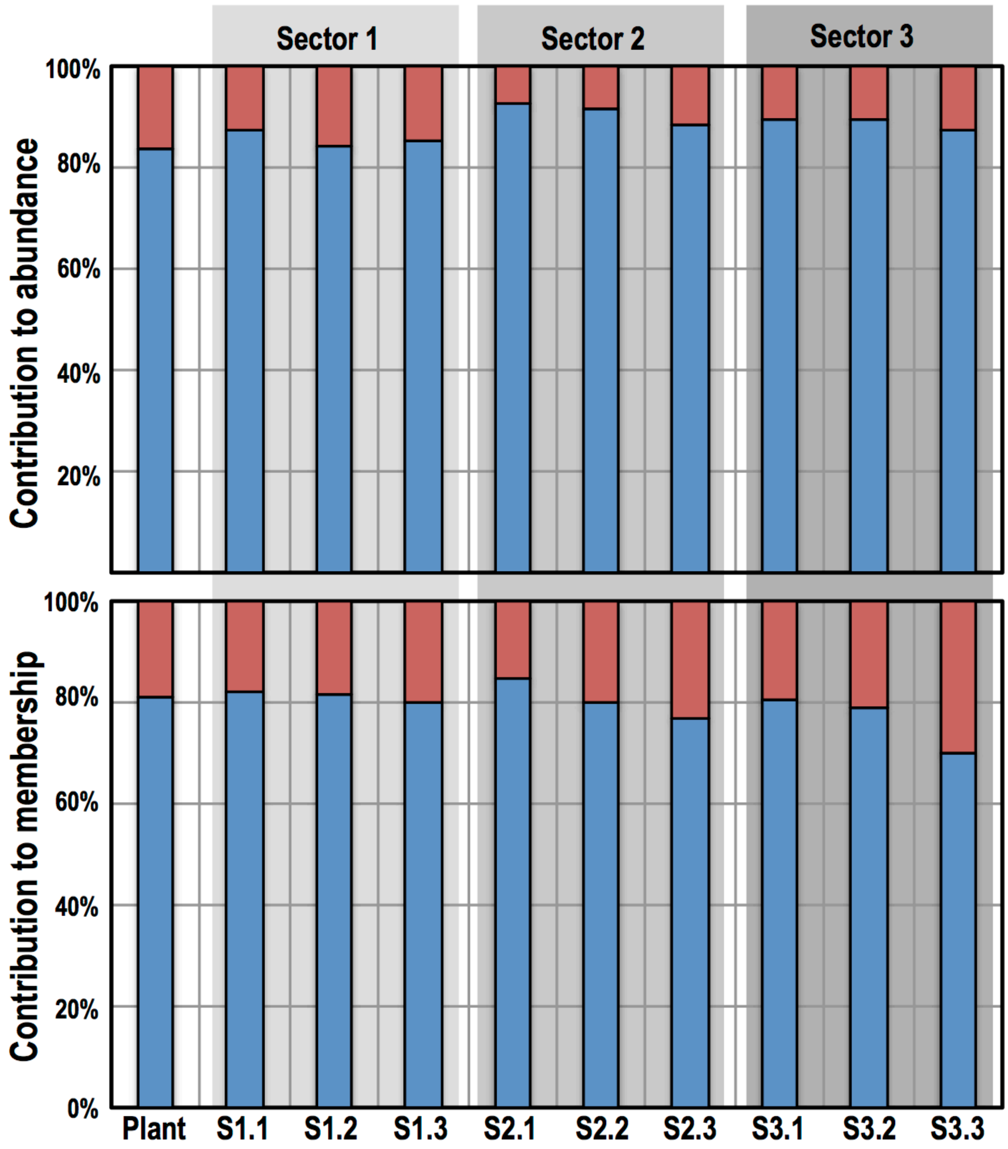

Supplement: Figure S2 — Contribution of site-specific (red) and non-site-specific (blue) OTUs to the relative abundance (top panel) and membership (bottom panel) at each sampling location for the entire sampling period. Download [file mbo003141850sf02.pdf]
