# Supplementary material for: Spatial-Temporal Survey and Occupancy-Abundance Modeling To Predict Bacterial Community Dynamics in the Drinking Water Microbiome
Source: mBio. 2014 May 27;5(3):e01135-14. doi: 10.1128/mBio.01135-14 (PMC4045074; doi:10.1128/mBio.01135-14)

**Supplementary Figure S3.** Effect of OTU filtering based on detection frequency (top panel) and relative abundance threshold (bottom panel) on Mantels r. The lower the Mantel r, the lower the correlation between the full dataset with all OTUs and the dataset with subset of OTUs based on detection frequency or relative abundance threshold.

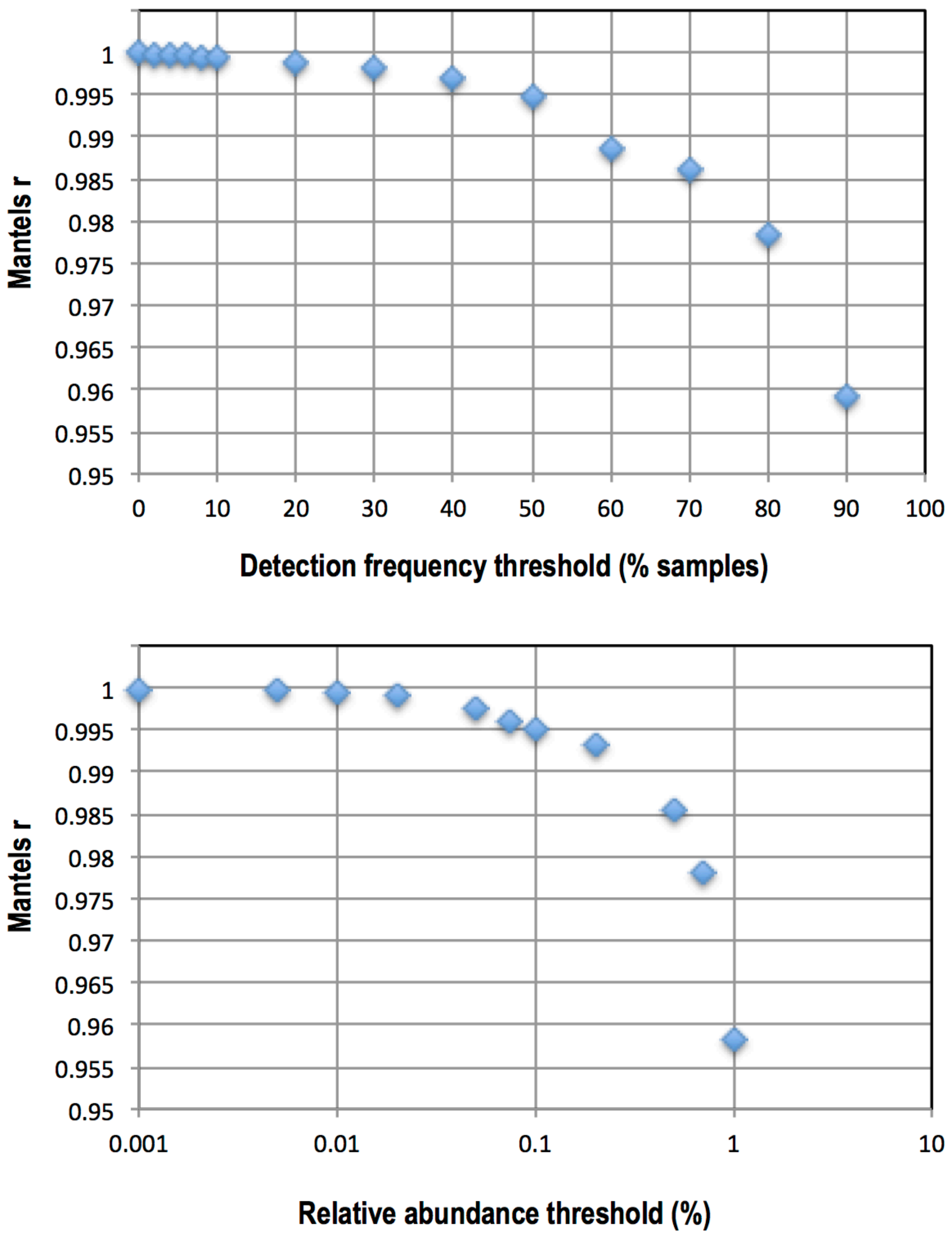

Supplement: Figure S3 — Effect of OTU filtering based on detection frequency (top panel) and relative abundance threshold (bottom panel) on Mantel’s r. The lower the Mantel r, the lower the correlation between the full data set with all OTUs and the data set with a subset of OTUs based on the detection frequency or relative abundance threshold. Download [file mbo003141850sf03.pdf]
