# Supplementary material for: Spatial-Temporal Survey and Occupancy-Abundance Modeling To Predict Bacterial Community Dynamics in the Drinking Water Microbiome
Source: mBio. 2014 May 27;5(3):e01135-14. doi: 10.1128/mBio.01135-14 (PMC4045074; doi:10.1128/mBio.01135-14)

Supplementary Figure S4. MIC associations between the three connected clusters are shown here. Isolated OTUs have been removed. (A) Green edges indicate positive MIC associations, while (B) red edges indicate negative MIC associations. These network visualisations clearly demonstrate within cluster positive associations and across cluster negative associations for the Cluster 1 (far right) and Cluster 2 (far left).

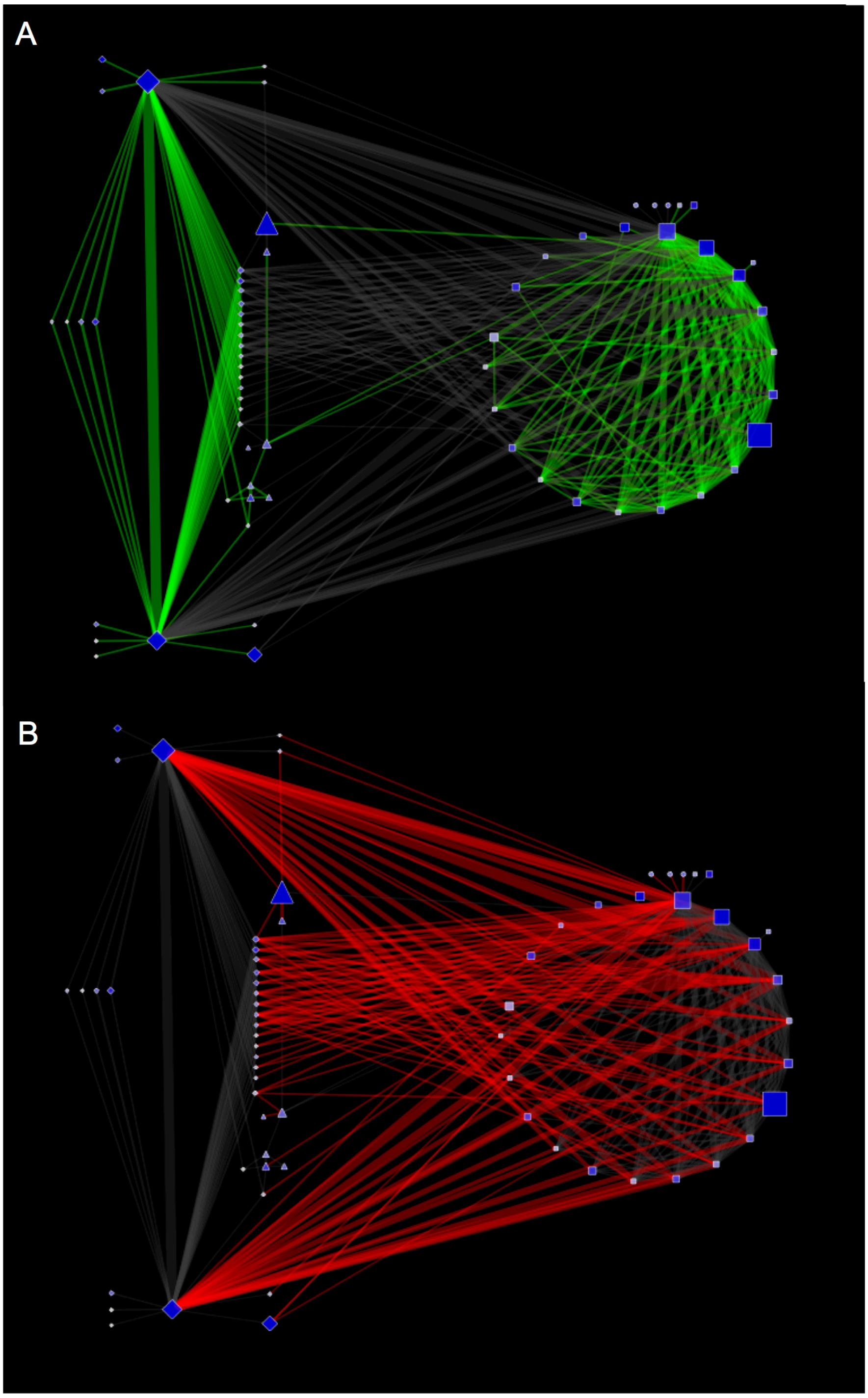

Supplement: Figure S4 — MIC associations between the three connected clusters are shown here. Isolated OTUs have been removed. Green edges indicate positive MIC associations (A), while red edges indicate negative MIC associations (B). These network visualizations are clearly demonstrated within cluster positive associations and across cluster negative associations for cluster 1 (far right) and cluster 2 (far left). Download [file mbo003141850sf04.pdf]
