# Supplementary material for: Spatial-Temporal Survey and Occupancy-Abundance Modeling To Predict Bacterial Community Dynamics in the Drinking Water Microbiome
Source: mBio. 2014 May 27;5(3):e01135-14. doi: 10.1128/mBio.01135-14 (PMC4045074; doi:10.1128/mBio.01135-14)
Supplement: Table S1 — Phylum-level classification of sequences detected at all sampling locations within each month shown as relative abundance (%). The phylum Proteobacteria is divided into its respective classes. [file mbo003141850st1.docx]

**Supplementary Table 1**. Phylum level classification of sequences detected at all sampling locations within each month shown as relative abundance (%). The phylum *Proteobacteria* is divided into its respective classes.

|  | | **2010** | | | | | | | **2011** | | | | | | | |
| --- | --- | --- | --- | --- | --- | --- | --- | --- | --- | --- | --- | --- | --- | --- | --- | --- |
|  | | **Jun** | **Jul** | **Aug** | **Sep** | **Oct** | **Nov** | **Dec** | **Jan** | **Feb** | **Mar** | **Apr** | **May** | **Jun** | **Jul** | **Aug** |
| *Proteobacteria* | *Alphaproteobacteria* | 14.5 | 16.57 | 20.6 | 25.29 | 20.34 | 26.62 | 36.77 | 33.16 | 27.31 | 15.11 | 15.8 | 11.07 | 10.45 | 11.15 | 12.84 |
|  | *Betaproteobacteria* | 41.9 | 41.23 | 57.37 | 45.92 | 48.31 | 22.3 | 25.32 | 28.38 | 33.95 | 30.44 | 37.95 | 50.6 | 58.05 | 58.33 | 53.52 |
|  | *Deltaproteobacteria* | 3.1 | 4.67 | 1.63 | 3.53 | 3.6 | 3.09 | 1.25 | 1.17 | 1.27 | 2.6 | 2.87 | 5.95 | 6.13 | 4 | 5.23 |
|  | *Epsilonproteobacteria* | <0.02 | <0.02 | 0 | 0 | <0.02 | 0 | 0 | 0 | <0.02 | <0.02 | <0.02 | <0.02 | 0 | <0.02 | <0.02 |
|  | *Gammaproteobacteria* | 2.18 | 3.01 | 2.01 | 2.27 | 3.84 | 8.71 | 6.22 | 7.84 | 13.17 | 11.06 | 23.39 | 9.76 | 1.37 | 1.54 | 1.57 |
|  | *Unclassified Proteobacteria* | 3.18 | 5.49 | 3.84 | 3.17 | 9.25 | 14.41 | 8.22 | 11.08 | 6.35 | 11.01 | 4.19 | 7.55 | 1.36 | 2.82 | 4.08 |
|  | *OD1* | 11.98 | 8.86 | 7.02 | 10.07 | 3.77 | 5.49 | 3.88 | 2.31 | 1.87 | 3.15 | 1.94 | 4.93 | 13.12 | 9.24 | 9.67 |
|  | *Acidobacteria* | 0.22 | 0.39 | 0.57 | 0.74 | 0.58 | 0.26 | 0.16 | 0.17 | 0.22 | 0.11 | 0.07 | 0.19 | 0.71 | 2.72 | 2.24 |
|  | *Chlamydiae* | 0.26 | 0.52 | 0.25 | 0.4 | 0.36 | 0.76 | 0.73 | 0.6 | 0.5 | 0.52 | 0.19 | 0.12 | 0.18 | 0.77 | 0.41 |
|  | *Planctomycetes* | 0.4 | 0.68 | 0.14 | 0.2 | 0.4 | 0.36 | 0.4 | 0.47 | 0.13 | 0.13 | 0.19 | 0.09 | 0.23 | 0.1 | 0.15 |
|  | *Bacteroidetes* | 0.21 | 0.37 | 0.17 | 0.24 | 0.26 | 0.21 | 0.09 | 0.17 | 0.14 | 0.28 | 0.29 | 0.18 | 0.22 | 0.31 | 0.33 |
|  | *Gemmatimonadetes* | 0.05 | 0.21 | 0.64 | 0.53 | 0.15 | 0.09 | 0.03 | <0.02 | 0.15 | 0.01 | 0.02 | 0.04 | 0.1 | 0.15 | 0.32 |
|  | *Actinobacteria* | 0.09 | 0.12 | 0.14 | 0.12 | 0.19 | 0.16 | 0.19 | 0.07 | 0.27 | 0.14 | 0.1 | 0.05 | 0.02 | 0.22 | 0.17 |
|  | *Verrucomicrobia* | 0.13 | 0.18 | 0.07 | 0.07 | 0.19 | 0.12 | 0.11 | 0.08 | 0.25 | 0.11 | 0.06 | 0.2 | 0.05 | 0.1 | 0.08 |
|  | *Firmicutes* | 0.22 | 0.12 | 0.06 | 0.07 | 0.17 | 0.25 | 0.09 | 0.06 | 0.12 | 0.08 | 0.05 | 0.15 | 0 | 0.03 | 0.02 |
|  | *Nitrospira* | 0.08 | 0.13 | 0.07 | 0.1 | 0.21 | 0.06 | 0.02 | 0 | 0.08 | 0.05 | <0.02 | 0.06 | 0.14 | 0.15 | 0.15 |
|  | *TM7* | 0.13 | 0.12 | 0.04 | 0.05 | 0.04 | 0.03 | 0.04 | 0.03 | <0.02 | 0.06 | 0.06 | 0.05 | <0.02 | 0.02 | 0.06 |
|  | *Chlorobi* | <0.02 | <0.02 | <0.02 | <0.02 | 0.02 | 0.1 | 0.03 | 0.03 | 0.07 | 0.12 | 0.02 | <0.02 | <0.02 | 0.02 | <0.02 |
|  | *Cyanobacteria_Chloroplast* | 0.02 | 0.1 | 0.02 | <0.02 | 0.03 | 0.17 | 0.03 | 0.02 | 0 | 0.02 | <0.02 | 0 | 0 | <0.02 | <0.02 |
|  | *Armatimonadetes* | <0.02 | 0.02 | <0.02 | <0.02 | 0.02 | 0.02 | <0.02 | 0.02 | 0 | 0 | 0 | <0.02 | 0.02 | 0.06 | 0.08 |
|  | *Chloroflexi* | <0.02 | <0.02 | <0.02 | <0.02 | 0.02 | 0.02 | <0.02 | 0 | 0.03 | <0.02 | 0 | 0 | <0.02 | <0.02 | 0 |
|  | *Spirochaetes* | 0 | 0.02 | <0.02 | <0.02 | <0.02 | 0.03 | <0.02 | 0.02 | <0.02 | 0 | <0.02 | 0 | <0.02 | <0.02 | <0.02 |
|  | *Deinococcus-Thermus* | <0.02 | <0.02 | <0.02 | <0.02 | <0.02 | 0 | 0 | 0 | 0 | <0.02 | 0 | 0 | 0 | <0.02 | 0.01 |
|  | *OP11* | <0.02 | <0.02 | 0 | <0.02 | <0.02 | <0.02 | 0 | 0 | 0 | 0 | 0 | <0.02 | 0 | 0 | 0 |
|  | *WS3* | <0.02 | <0.02 | 0 | <0.02 | <0.02 | 0 | 0 | 0 | 0 | <0.02 | 0 | 0 | <0.02 | 0 | 0 |
|  | *Tenericutes* | <0.02 | 0 | 0 | 0 | 0 | 0 | 0 | 0 | 0 | 0 | 0 | 0 | 0 | 0 | 0 |
