# Supplementary material for: Spatial-Temporal Survey and Occupancy-Abundance Modeling To Predict Bacterial Community Dynamics in the Drinking Water Microbiome
Source: mBio. 2014 May 27;5(3):e01135-14. doi: 10.1128/mBio.01135-14 (PMC4045074; doi:10.1128/mBio.01135-14)
Supplement: Table S2 — Water quality data for all sampling locations and time points. [file mbo003141850st2.docx]

**Table S2:** Water quality data for all sampling locations and timepoints

| **Location** | **Month** | **Total organic carbon** | **Ammonia** | **Phosphorus** | **pH** | **Nitrate** | **Turbidity** | **Conductivity** | **Temperature** | **Chloride** | **Sulfate** | **Total chlorine** |
| --- | --- | --- | --- | --- | --- | --- | --- | --- | --- | --- | --- | --- |
|  |  | **ppm as C** | **ppb as N** | **ppb as P** |  | **ppb as N** | **NTU** | **microS/cm** | **degree C** | **ppm** | **ppm** | **ppm as Cl2** |
| Plant | Jun-10 | 4.62 | 321.9 | 73.6 | 9.25 | 1292.5 | 0.02 | 0.44 | 21.2 | 118.9 | 14.3 | 2.98 |
| S1.2 | Jun-10 | 5.31 | 414.2 | 173.8 | 8.96 | 1546.4 | 0.2 | 0.49 | 20.1 | 122.5 | 15.0 | 2.40 |
| S1.3 | Jun-10 | 4.52 | 481.0 | 143.6 | 8.97 | 1209.6 | 0.2 | 0.49 | 18.8 | 112.9 | 13.2 | 2.00 |
| S2.1 | Jun-10 | 4.69 | 370.1 | 129.6 | 9.04 | 918.0 | 0.22 | 0.49 | 19 | 549.0 | 16.2 | 2.30 |
| S2.2 | Jun-10 | 5.29 | 355.6 | 152.2 | 9.06 | 1282.5 | 0.14 | 0.54 | 17.7 | 119.1 | 14.9 | 2.70 |
| S2.3 | Jun-10 | 5.61 | 284.1 | 122.1 | 9.02 | 1352.8 | 0.13 | 0.55 | 19.3 | 732.1 | 20.2 | 2.80 |
| S3.1 | Jun-10 | 5.54 | 303.9 | 143.4 | 9.14 | 923.0 | 0.18 | 0.54 | 19.9 | 614.3 | 18.1 | 2.40 |
| S3.2 | Jun-10 | 5.14 | 388.7 | 122.2 | 9.07 | 1380.5 | 0.17 | 0.53 | 20.2 | 106.9 | 13.7 | 2.60 |
| S3.3 | Jun-10 | 4.64 | 459.5 | 166.5 | 9.13 | 1739.9 | 0.16 | 0.51 | 19.1 | 109.4 | 13.3 | 2.20 |
| Plant | Jul-10 | 3.65 | 232.3 | 153.2 | 9.20 | 0.0 | 0.04 | 0.48 | 23.1 | 25.5 | 6.3 | 3.10 |
| S1.1 | Jul-10 | 3.69 | 168.8 | 252.5 | 9.33 | 0.0 | 0.56 | 0.48 | 26.6 | 45.3 | 6.8 | 2.90 |
| S1.2 | Jul-10 | 3.56 | 320.9 | 143.1 | 9.39 | 358.9 | 0.17 | 0.46 | 25.2 | 52.6 | 9.0 | 2.70 |
| S1.3 | Jul-10 | 3.88 | 361.5 | 162.7 | 9.25 | 240.1 | 0.29 | 0.48 | 22 | 44.3 | 8.2 | 2.30 |
| S2.1 | Jul-10 | 3.47 | 286.5 | 167.2 | 9.28 | 540.1 | 0.26 | 0.48 | 23.1 | 48.2 | 8.6 | 2.50 |
| S2.2 | Jul-10 | 4.76 | 326.1 | 181.4 | 9.06 | 193.2 | 0.15 | 0.45 | 22.7 | 50.6 | 8.8 | 2.70 |
| S2.3 | Jul-10 | 3.39 | 298.0 | 199.3 | 9.30 | 236.9 | 0.22 | 0.46 | 26.2 | 50.5 | 8.9 | 2.80 |
| S3.1 | Jul-10 | 3.64 | 258.4 | 219.6 | 9.24 | 521.4 | 0.44 | 0.46 | 23.7 | 43.4 | 8.2 | 2.50 |
| S3.2 | Jul-10 | 4.57 | 312.6 | 204.4 | 9.17 | 780.8 | 0.4 | 0.46 | 23.2 | 41.3 | 8.0 | 3.00 |
| S3.3 | Jul-10 | 3.69 | 309.4 | 282.3 | 9.47 | 580.8 | 0.23 | 0.47 | 21.2 | 43.4 | 8.1 | 2.80 |
| Plant | Aug-10 | 3.93 | 321.0 | 81.9 | 9.30 | 359.5 | 0.04 | 0.48 | 23.6 | 62.6 | 12.9 | 3.00 |
| S1.1 | Aug-10 | 3.02 | 288.3 | 131.7 | 9.30 | 0.0 | 0.16 | 0.45 | 25.8 | 54.3 | 12.5 | 2.90 |
| S1.2 | Aug-10 | 3.31 | 328.8 | 129.1 | 9.30 | 138.2 | 0.17 | 0.46 | 24.3 | 46.1 | 10.5 | 2.60 |
| S1.3 | Aug-10 | 3.13 | 391.9 | 124.7 | 9.30 | 149.9 | 0.16 | 0.48 | 23.3 | 75.9 | 14.2 | 2.00 |
| S2.1 | Aug-10 | 2.53 | 408.8 | 133.3 | 9.30 | 0.0 | 0.21 | 0.48 | 23.6 | 44.5 | 10.1 | 2.40 |
| S2.2 | Aug-10 | 2.71 | 292.8 | 34.7 | 9.30 | 0.0 | 0.2 | 0.46 | 22 | 55.0 | 11.3 | 2.50 |
| S2.3 | Aug-10 | 2.75 | 317.6 | 40.6 | 9.20 | 0.0 | 0.13 | 0.46 | 25.6 | 60.8 | 10.6 | 2.80 |
| S3.1 | Aug-10 | 2.95 | 362.6 | 80.9 | 9.30 | 367.2 | 3.2 | 0.46 | 24.6 | 60.5 | 12.4 | 2.40 |
| S3.2 | Aug-10 | 3.15 | 340.1 | 125.8 | 9.30 | 178.3 | 0.12 | 0.46 | 23.9 | 54.1 | 11.6 | 3.00 |
| S3.3 | Aug-10 | 3.34 | 541.7 | 108.0 | 9.40 | 421.6 | 0.35 | 0.49 | 22.7 | 57.8 | 12.2 | 2.50 |
| Plant | Sep-10 | 2.15 | 285.0 | 145.2 | 9.48 | 1179.7 | 0.02 | 0.56 | 19.7 | 70.1 | 15.9 | 3.00 |
| S1.1 | Sep-10 | 2.71 | 167.9 | 120.8 | 9.20 | 1854.7 | 0.23 | 0.56 | 22.1 | 138.2 | 23.0 | 2.90 |
| S1.2 | Sep-10 | 2.9 | 160.3 | 177.2 | 9.20 | 731.4 | 0.13 | 0.55 | 22 | 103.8 | 19.4 | 2.70 |
| S2.1 | Sep-10 | 2.67 | 334.9 | 103.5 | 9.30 | 849.6 | 0.12 | 0.56 | 21.9 | 79.7 | 18.4 | 2.40 |
| S2.2 | Sep-10 | 2.9 | 208.2 | 100.8 | 9.20 | 0.0 | 0.18 | 0.55 | 21.2 | 38.1 | 11.1 | 2.80 |
| S2.3 | Sep-10 | 2.79 | 143.0 | 133.3 | 9.20 | 0.0 | 0.13 | 0.55 | 22.6 | 535.0 | 26.9 | 2.70 |
| S3.1 | Sep-10 | 2.62 | 185.2 | 108.7 | 9.20 | 283.0 | 0.17 | 0.57 | 22.4 | 139.6 | 29.6 | 2.20 |
| S3.2 | Sep-10 | 2.53 | 227.4 | 83.2 | 9.30 | 632.8 | 0.1 | 0.56 | 20.9 | 86.6 | 18.7 | 2.90 |
| S3.3 | Sep-10 | 3.07 | 177.5 | 65.2 | 9.40 | 228.8 | 0.16 | 0.57 | 21.6 | 86.4 | 17.4 | 2.50 |
| Plant | Oct-10 | 4.66 | 312.6 | 77.7 | 9.16 | 573.6 | 0.05 | 0.65 | 14.5 | 121.6 | 31.6 | 3.00 |
| S1.1 | Oct-10 | 5.08 | 303.1 | 82.0 | 9.30 | 0.0 | 0.1 | 0.62 | 21.6 | 93.1 | 24.8 | 2.90 |
| S1.2 | Oct-10 | 5.06 | 257.1 | 110.3 | 9.30 | 203.1 | 0.09 | 0.62 | 17.5 | 86.7 | 23.1 | 2.80 |
| S1.3 | Oct-10 | 3.84 | 268.6 | 82.5 | 9.30 | 0.0 | 0.16 | 0.64 | 18.3 | 100.9 | 24.6 | 2.10 |
| S2.1 | Oct-10 | 5.34 | 285.8 | 92.1 | 9.30 | 0.0 | 0.11 | 0.63 | 18.6 | 144.8 | 31.5 | 2.80 |
| S2.2 | Oct-10 | 4.97 | 291.6 | 181.1 | 9.30 | 0.0 | 0.07 | 0.62 | 19.3 | 90.8 | 23.6 | 2.90 |
| S2.3 | Oct-10 | 4.17 | 463.8 | 112.0 | 9.30 | 0.0 | 0.11 | 0.62 | 19.5 | 67.5 | 19.4 | 3.00 |
| S3.1 | Oct-10 | 5.06 | 433.2 | 77.9 | 9.40 | 0.0 | 0.15 | 0.63 | 18.6 | 100.9 | 25.3 | 2.20 |
| S3.2 | Oct-10 | 4.66 | 285.8 | 82.6 | 9.40 | 0.0 | 0.08 | 0.63 | 17.8 | 141.4 | 29.5 | 2.70 |
| S3.3 | Oct-10 | 4.61 | 389.2 | 76.0 | 9.50 | 0.0 | 0.12 | 0.65 | 18.7 | 131.8 | 30.2 | 2.30 |
| Plant | Nov-10 | 3.44 | 309.7 | 40.1 | 9.24 | 562.7 | 0.03 | 0.8 | 10.5 | 216.7 | 35.7 | 3.00 |
| S1.2 | Nov-10 | 3.7 | 494.5 | 243.0 | 9.20 | 633.0 | 0.18 | 0.61 | 16.3 | 130.3 | 36.1 | 2.60 |
| S1.3 | Nov-10 | 3.2 | 390.5 | 71.8 | 9.20 | 588.2 | 0.22 | 0.62 | 16.5 | 138.6 | 34.8 | 2.20 |
| S2.1 | Nov-10 | 3.3 | 432.4 | 89.8 | 9.30 | 572.3 | 0.24 | 0.62 | 14.9 | 131.3 | 35.5 | 2.50 |
| S2.2 | Nov-10 | 3.77 | 515.5 | 159.0 | 9.10 | 601.0 | 0.1 | 0.61 | 16.8 | 131.4 | 36.0 | 3.00 |
| S3.1 | Nov-10 | 3.31 | 556.7 | 46.0 | 9.20 | 620.2 | 0.22 | 0.64 | 12.7 | 138.1 | 36.8 | 2.60 |
| S3.2 | Nov-10 | 3.59 | 499.2 | 18.7 | 9.20 | 629.8 | 0.22 | 0.66 | 13.1 | 139.8 | 33.6 | 2.90 |
| S3.3 | Nov-10 | 3.12 | 441.7 | 101.8 | 9.40 | 591.4 | 0.44 | 0.62 | 15.4 | 131.3 | 35.1 | 2.70 |
| Plant | Dec-10 | 2.28 | 481.8 | 122.3 | 9.02 | 541.6 | 0.04 | 0.81 | 8.1 | 244.8 | 33.4 | 3.00 |
| S1.1 | Dec-10 | 2.1 | 444.5 | 152.1 | 9.30 | 504.6 | 0.16 | 0.81 | 13.6 | 245.5 | 32.7 | 2.60 |
| S1.2 | Dec-10 | 2.09 | 462.7 | 269.3 | 9.40 | 464.3 | 0.14 | 0.83 | 10.6 | 288.6 | 37.0 | 2.70 |
| S2.1 | Dec-10 | 2.38 | 446.8 | 134.4 | 9.30 | 548.3 | 0.3 | 0.82 | 10.7 | 243.9 | 33.0 | 2.50 |
| S2.2 | Dec-10 | 2.15 | 427.8 | 230.7 | 9.30 | 501.3 | 0.43 | 0.83 | 16.1 | 253.1 | 34.1 | 2.60 |
| S2.3 | Dec-10 | 2.17 | 465.1 | 163.0 | 9.40 | 555.1 | 0.14 | 0.83 | 11.5 | 244.7 | 33.5 | 2.60 |
| S3.1 | Dec-10 | 2.48 | 497.7 | 123.7 | 9.30 | 464.3 | 0.32 | 0.81 | 10.6 | 272.8 | 35.3 | 2.40 |
| S3.2 | Dec-10 | 2.44 | 460.3 | 141.9 | 9.30 | 541.6 | 0.26 | 0.82 | 11.3 | 248.3 | 33.2 | 2.60 |
| S3.3 | Dec-10 | 2.44 | 433.3 | 131.2 | 9.50 | 585.3 | 0.34 | 0.81 | 11.6 | 234.5 | 31.2 | 2.50 |
| Plant | Jan-11 | 2.37 | 496.9 | 287.0 | 8.52 | 650.1 | 0.08 | 0.76 | 7.8 | 115.4 | 24.3 | 3.00 |
| S1.1 | Jan-11 | 2.82 | 562.0 | 152.4 | 8.70 | 420.6 | 0.12 | 0.75 | 8.9 | 88.8 | 21.1 | 2.40 |
| S1.2 | Jan-11 | 3.13 | 457.2 | 208.8 | 8.80 | 474.8 | 0.13 | 0.75 | 10.6 | 74.5 | 19.6 | 2.50 |
| S2.2 | Jan-11 | 2.66 | 520.7 | 109.2 | 8.80 | 347.3 | 0.09 | 0.75 | 8.5 | 60.4 | 18.5 | 2.50 |
| S2.3 | Jan-11 | 2.83 | 374.5 | 98.1 | 8.80 | 423.8 | 0.05 | 0.75 | 7.9 | 87.8 | 21.5 | 2.70 |
| S3.2 | Jan-11 | 2.68 | 432.5 | 124.1 | 9.00 | 344.1 | 0.07 | 0.76 | 7.6 | 77.2 | 20.0 | 2.40 |
| S3.3 | Jan-11 | 2.97 | 367.4 | 171.9 | 9.20 | 360.0 | 0.15 | 0.76 | 7.5 | 78.8 | 20.0 | 2.00 |
| Plant | Feb-11 | 3.64 | 359.7 | 108.6 | 8.92 | 609.1 | 0.32 | 0.75 | 8.2 | 161.8 | 27.4 | 3.00 |
| S1.2 | Feb-11 | 3.55 | 396.7 | 160.2 | 8.84 | 796.8 | 0.15 | 0.73 | 5.5 | 157.8 | 26.8 | 2.40 |
| S1.3 | Feb-11 | 3.28 | 382.7 | 157.8 | 9.43 | 975.0 | 0.58 | 0.75 | 9 | 163.9 | 26.9 | 2.00 |
| S2.1 | Feb-11 | 3.25 | 364.6 | 164.2 | 8.82 | 669.5 | 0.4 | 0.75 | 7.2 | 160.2 | 27.1 | 2.30 |
| S2.2 | Feb-11 | 3.56 | 335.9 | 123.3 | 8.86 | 500.9 | 0.14 | 0.74 | 11.3 | 157.0 | 27.1 | 2.70 |
| S2.3 | Feb-11 | 3.48 | 348.2 | 137.5 | 8.82 | 873.2 | 0.09 | 0.74 | 6.5 | 157.1 | 26.8 | 2.00 |
| S3.1 | Feb-11 | 3.33 | 386.0 | 137.0 | 9.45 | 736.3 | 0.42 | 0.75 | 7 | 161.5 | 27.2 | 2.10 |
| S3.2 | Feb-11 | 3.36 | 343.3 | 131.5 | 9.45 | 570.9 | 0.49 | 0.74 | 6.8 | 162.3 | 27.3 | 2.70 |
| S3.3 | Feb-11 | 3.49 | 374.5 | 202.8 | 9.54 | 914.5 | 0.35 | 0.75 | 6.1 | 162.2 | 27.3 | 2.30 |
| Plant | Mar-11 | 3.67 | 286.1 | 126.0 | 8.92 | 831.5 | 0.05 | 0.72 | 5 | 178.1 | 45.6 | 3.00 |
| S1.1 | Mar-11 | 3.89 | 390.7 | 169.8 | 8.83 | 996.3 | 0.16 | 0.75 | 11.9 | 181.6 | 44.9 | 2.70 |
| S1.2 | Mar-11 | 3.49 | 382.5 | 174.5 | 8.79 | 842.5 | 0.11 | 0.75 | 7.8 | 181.8 | 44.8 | 2.40 |
| S1.3 | Mar-11 | 3.77 | 314.5 | 223.6 | 8.85 | 1022.0 | 0.28 | 0.75 | 6.5 | 190.9 | 46.7 | 2.50 |
| S2.1 | Mar-11 | 4.87 | 377.3 | 192.6 | 8.66 | 1062.3 | 0.22 | 0.76 | 5.7 | 190.4 | 45.4 | 2.40 |
| S2.2 | Mar-11 | 2.68 | 413.9 | 182.8 | 8.63 | 871.8 | 0.17 | 0.74 | 8.2 | 182.3 | 44.8 | 2.50 |
| S2.3 | Mar-11 | 3.83 | 341.4 | 258.4 | 8.67 | 776.6 | 0.09 | 0.75 | 6.4 | 182.9 | 45.4 | 2.90 |
| S3.1 | Mar-11 | 3.82 | 397.4 | 176.8 | 8.87 | 868.1 | 0.35 | 0.71 | 4.9 | 183.8 | 45.0 | 2.40 |
| S3.2 | Mar-11 | 3.38 | 395.2 | 185.1 | 8.77 | 824.2 | 0.1 | 0.77 | 5.4 | 181.1 | 44.7 | 2.50 |
| S3.3 | Mar-11 | 2.77 | 372.0 | 187.4 | 8.93 | 732.6 | 0.13 | 0.76 | 7 | 183.5 | 44.9 | 2.50 |
| Plant | Apr-11 | 4.24 | 330.0 | 518.8 | 8.46 | 796.3 | 0.07 | 0.65 | 11 | 158.4 | 61.5 | 3.00 |
| S1.1 | Apr-11 | 4.22 | 361.7 | 490.5 | 8.41 | 698.1 | 0.18 | 0.6 | 18.6 | 161.4 | 62.2 | 2.50 |
| S1.2 | Apr-11 | 3.34 | 379.5 | 299.5 | 8.60 | 752.7 | 0.15 | 0.65 | 11.8 | 160.6 | 61.3 | 2.50 |
| S1.3 | Apr-11 | 3.64 | 358.6 | 207.4 | 8.76 | 759.9 | 0.25 | 0.66 | 13.9 | 165.9 | 61.6 | 2.50 |
| S2.1 | Apr-11 | 3.39 | 343.1 | 277.8 | 8.69 | 712.6 | 0.16 | 0.65 | 10.5 | 161.9 | 61.5 | 2.20 |
| S2.2 | Apr-11 | 4.1 | 361.7 | 225.7 | 8.73 | 829.1 | 0.26 | 0.64 | 13.1 | 158.8 | 61.3 | 2.80 |
| S2.3 | Apr-11 | 3.81 | 328.4 | 199.8 | 8.77 | 945.6 | 0.18 | 0.64 | 16.3 | 159.3 | 61.1 | 2.80 |
| S3.1 | Apr-11 | 4.03 | 364.8 | 200.9 | 8.69 | 745.4 | 0.48 | 0.64 | 9.9 | 159.2 | 61.3 | 2.00 |
| S3.2 | Apr-11 | 3.74 | 325.3 | 155.1 | 8.81 | 701.7 | 0.17 | 0.64 | 11.3 | 160.6 | 61.2 | 2.60 |
| S3.3 | Apr-11 | 3.78 | 368.7 | 256.6 | 8.91 | 876.4 | 0.26 | 0.66 | 10.2 | 163.7 | 61.6 | 2.00 |
| Plant | May-11 | 3.95 | 299.0 | 272.1 | 8.90 | 625.3 | 0.05 | 0.62 | 19.6 | 178.1 | 47.2 | 2.90 |
| S1.1 | May-11 | 4.42 | 371.0 | 212.5 | 8.61 | 534.3 | 0.17 | 0.61 | 20.3 | 181.6 | 44.9 | 2.60 |
| S1.2 | May-11 | 4.29 | 333.1 | 185.2 | 9.00 | 443.3 | 0.13 | 0.6 | 18.9 | 181.8 | 44.8 | 2.40 |
| S1.3 | May-11 | 4.3 | 381.9 | 186.0 | 8.99 | 738.1 | 0.23 | 0.63 | 17.7 | 190.9 | 46.7 | 1.80 |
| S2.1 | May-11 | 4.54 | 326.9 | 198.4 | 9.04 | 516.1 | 0.24 | 0.6 | 14.6 | 190.4 | 45.4 | 2.50 |
| S2.2 | May-11 | 4.4 | 320.7 | 167.9 | 9.02 | 636.2 | 0.21 | 0.59 | 14.4 | 182.3 | 44.8 | 2.60 |
| S2.3 | May-11 | 4.5 | 319.9 | 174.3 | 9.01 | 476.1 | 0.18 | 0.6 | 20.7 | 182.9 | 45.4 | 2.70 |
| S3.1 | May-11 | 4.45 | 434.5 | 149.8 | 8.94 | 516.1 | 0.41 | 0.62 | 14.3 | 183.8 | 45.0 | 2.40 |
| S3.2 | May-11 | 3.77 | 230.9 | 145.6 | 9.32 | 545.2 | 0.13 | 0.64 | 15.3 | 181.1 | 44.7 | 2.60 |
| S3.3 | May-11 | 4.1 | 371.0 | 201.2 | 8.82 | 643.5 | 0.19 | 0.63 | 14.3 | 183.5 | 44.9 | 2.40 |
| Plant | Jun-11 | 4.63 | 314.9 | 204.3 | 9.26 | 569.2 | 0.04 | 0.56 | 22.6 | 149.8 | 77.4 | 2.90 |
| S1.1 | Jun-11 | 4.77 | 248.6 | 195.0 | 8.95 | 453.9 | 0.09 | 0.54 | 21 | 157.8 | 80.6 | 2.80 |
| S1.2 | Jun-11 | 5.55 | 324.6 | 209.5 | 9.17 | 577.2 | 0.11 | 0.55 | 25 | 145.4 | 74.7 | 2.40 |
| S1.3 | Jun-11 | 4.54 | 383.9 | 240.9 | 9.26 | 537.4 | 0.14 | 0.55 | 21 | 147.8 | 73.9 | 2.20 |
| S2.2 | Jun-11 | 5.37 | 334.8 | 209.8 | 9.22 | 362.4 | 0.14 | 0.55 | 23.6 | 148.8 | 74.8 | 2.50 |
| S2.3 | Jun-11 | 4.86 | 357.5 | 232.0 | 9.22 | 529.4 | 0.09 | 0.54 | 25.1 | 149.7 | 73.9 | 2.70 |
| S3.2 | Jun-11 | 5.24 | 459.9 | 235.9 | 9.28 | 386.2 | 0.06 | 0.54 | 20.6 | 157.1 | 75.7 | 2.70 |
| S3.3 | Jun-11 | 4.13 | 328.9 | 235.2 | 9.26 | 489.7 | 0.13 | 0.55 | 20.9 | 148.6 | 75.6 | 2.30 |
| Plant | Jul-11 | 4.15 | 148.3 | 212.2 | 9.26 | 816.8 | 0.06 | 0.5 | 22.6 | 106.8 | 30.1 | 3.00 |
| S1.1 | Jul-11 | 3.92 | 123.3 | 216.9 | 8.86 | 292.8 | 0.07 | 0.55 | 26.7 | 152.5 | 29.7 | 3.10 |
| S1.2 | Jul-11 | 3.81 | 89.2 | 225.5 | 9.09 | 316.4 | 0.08 | 0.55 | 26.7 | 153.1 | 29.7 | 2.80 |
| S1.3 | Jul-11 | 4.08 | 99.5 | 217.0 | 9.32 | 344.0 | 0.13 | 0.55 | 24.3 | 144.0 | 33.6 | 2.60 |
| S2.1 | Jul-11 | 3.98 | 117.5 | 228.1 | 9.19 | 388.5 | 0.1 | 0.55 | 25.8 | 150.8 | 30.8 | 2.60 |
| S2.2 | Jul-11 | 3.32 | 102.5 | 204.3 | 9.18 | 320.3 | 0.08 | 0.55 | 25.8 | 151.6 | 29.4 | 2.90 |
| S2.3 | Jul-11 | 3.76 | 127.3 | 210.7 | 9.15 | 304.6 | 0.09 | 0.55 | 28.9 | 151.4 | 29.2 | 3.00 |
| S3.2 | Jul-11 | 3.66 | 90.9 | 237.3 | 9.25 | 355.8 | 0.12 | 0.56 | 24.6 | 152.9 | 28.9 | 3.00 |
| S3.3 | Jul-11 | 3.89 | 87.2 | 200.5 | 9.33 | 434.6 | 0.12 | 0.57 | 24 | 144.3 | 31.9 | 2.50 |
| Plant | Aug-11 | 5.17 | 320.4 | 392.4 | 9.40 | 161.2 | 0.05 | 0.57 | 26.5 | 123.0 | 29.0 | 3.00 |
| S1.1 | Aug-11 | 5.39 | 378.5 | 244.9 | 9.30 | 186.8 | 0.1 | 0.53 | 22.8 | 113.4 | 27.8 | 3.00 |
| S1.2 | Aug-11 | 4.8 | 426.9 | 278.9 | 9.30 | 355.3 | 0.1 | 0.56 | 24.3 | 128.9 | 29.3 | 2.40 |
| S1.3 | Aug-11 | 4.89 | 315.6 | 266.9 | 9.20 | 234.4 | 0.11 | 0.54 | 23.7 | 125.6 | 28.6 | 2.00 |
| S2.1 | Aug-11 | 4.11 | 384.8 | 256.5 | 9.30 | 329.7 | 0.11 | 0.53 | 24.6 | 124.7 | 28.5 | 2.20 |
| S2.2 | Aug-11 | 4.69 | 407.2 | 250.8 | 9.30 | 274.7 | 0.09 | 0.56 | 22.7 | 118.9 | 28.3 | 2.50 |
| S2.3 | Aug-11 | 4.65 | 434.9 | 249.7 | 9.30 | 391.9 | 0.08 | 0.56 | 24.2 | 128.3 | 29.6 | 2.60 |
| S3.1 | Aug-11 | 4.74 | 274.1 | 232.0 | 9.20 | 223.4 | 0.21 | 0.55 | 26.1 | 127.7 | 29.1 | 1.80 |
| S3.2 | Aug-11 | 5.36 | 417.3 | 260.7 | 9.30 | 223.4 | 0.18 | 0.55 | 24.3 | 125.8 | 28.4 | 2.60 |
| S3.3 | Aug-11 | 4.75 | 465.3 | 268.4 | 9.40 | 304.0 | 0.14 | 0.56 | 23.2 | 128.3 | 29.3 | 2.40 |
