# Supplementary material for: Spatial-Temporal Survey and Occupancy-Abundance Modeling To Predict Bacterial Community Dynamics in the Drinking Water Microbiome
Source: mBio. 2014 May 27;5(3):e01135-14. doi: 10.1128/mBio.01135-14 (PMC4045074; doi:10.1128/mBio.01135-14)
Supplement: Table S3 — (A) Summary of PERMANOVA results indicating the explanatory power of temporal and seasonal factors with respect to bacterial community structure. The top table compares the month and location, while the bottom table compares the season and sector for all four beta diversity metrics. (B) Global ANOSIM summaries and respective significance values for comparing samples grouped by month, location, season, and sector. [file mbo003141850st3.docx]

**Supplementary Table 3: (A)** Summary of PERMANOVA results indicating the explanatory power of temporal and seasonal factors with respect to bacterial community structure. Top table compares the month vs location, while bottom table compares season vs sector for all four beta-diversity metrics. (B) Global ANOSIM summaries and respective significance values for comparing samples grouped by month, location, season, and sector.

**Table 3A**

|  | Degrees of freedom | Sum of squares | Mean square | F.model | R2 | Pr(>F) |
| --- | --- | --- | --- | --- | --- | --- |
| **Community membership based metrics** | | | | | | |
| Jaccard distance | | | | | | |
| Month | 14 | 11.42 | 0.82 | 3.63 | 0.29 | 0.001 |
| Location | 9 | 2.93 | 0.33 | 1.45 | 0.07 | 0.001 |
| Residuals | 114 | 25.63 | 0.22 |  | 0.64 |  |
| Total | 137 | 39.99 |  |  |  |  |
| Unweighted Unifrac distance | | | | | | |
| Month | 14 | 9.24 | 0.66 | 3.60 | 0.28 | 0.001 |
| Location | 9 | 2.47 | 0.27 | 1.50 | 0.08 | 0.001 |
| Residuals | 114 | 20.92 | 0.1 |  | 0.64 |  |
| Total | 137 | 32.63 |  |  |  |  |
| **Community structure based metrics** | | | | | | |
| Bray-Curtis distance | | | | | | |
| Month | 14 | 15.13 | 1.08 | 23.93 | 0.71 | 0.001 |
| Location | 9 | 0.98 | 0.109 | 2.41 | 0.05 | 0.001 |
| Residuals | 114 | 5.15 | 0.045 | 0.24 |  |  |
| Total | 137 | 21.27 |  |  |  |  |
| Weighted Unifrac distance | | | | | | |
| Month | 14 | 6.45 | 0.46 | 20.79 | 0.67 | 0.001 |
| Location | 9 | 0.62 | 0.069 | 3.10 | 0.07 | 0.001 |
| Residuals | 114 | 2.53 | 0.022 | 0.26 |  |  |
| Total | 137 | 9.60 |  |  |  |  |

|  | Degrees of freedom | Sum of squares | Mean square | F.model | R2 | Pr(>F) |
| --- | --- | --- | --- | --- | --- | --- |
| **Community membership based metrics** | | | | | | |
| Jaccard distance | | | | | | |
|  | Df | SumsOfSqs | MeanSqs | F.Model | R2 | Pr(>F) |
| Season | 4 | 6.45 | 1.61 | 6.46 | 0.16 | 0.001 |
| Sector | 3 | 1.12 | 0.37 | 1.50 | 0.028 | 0.002 |
| Residuals | 130 | 32.43 | 0.25 | 0.81 |  |  |
| Unweighted Unifrac distance | | | | | | |
|  | Df | SumsOfSqs | MeanSqs | F.Model | R2 | Pr(>F) |
| Season | 4 | 5.25 | 1.31 | 6.45 | 0.16 | 0.001 |
| Sector | 3 | 0.96 | 0.32 | 1.57 | 0.03 | 0.001 |
| Residuals | 130 | 26.43 | 0.20 | 0.81 |  |  |
| **Community structure based metrics** | | | | | | |
| Bray-Curtis distance | | | | | | |
| Season | 4 | 10.33 | 2.58 | 31.75 | 0.49 | 0.001 |
| Sector | 3 | 0.36 | 0.12 | 1.47 | 0.02 | 0.104 |
| Residuals | 130 | 10.58 | 0.08 | 0.50 |  |  |
| Total | 137 | 21.27 | 1 |  |  |  |
| Weighted Unifrac distance | | | | | | |
| Season | 4 | 4.27 | 1.07 | 27.29 | 0.44 | 0.001 |
| Sector | 3 | 0.24 | 0.08 | 2.01 | 0.02 | 0.019 |
| Residuals | 130 | 5.01 | 0.039 | 0.53 |  |  |
| Total | 137 | 9.60 | 1 |  |  |  |

**Table 3B.**

|  | ANOSIM Global R | Significance |
| --- | --- | --- |
| **Community membership based metrics** | | |
| **Jaccard distance** |  |  |
| Month | 0.87 | 0.001 |
| Location | 0.05 | 0.015 |
| Season | 0.74 | 0.001 |
| Sector | 0.03 | 0.046 |
| **Unweighted Unifrac** |  |  |
| Month | 0.79 | 0.001 |
| Location | 0.05 | 0.006 |
| Season | 0.66 | 0.001 |
| Sector | 0.02 | 0.122 |
| **Community structure based metrics** | | |
| **Bray-Curtis distance** | | |
| Month | 0.90 | 0.001 |
| Location | 0 | 0.94 |
| Season | 0.70 | 0.001 |
| Sector | 0 | 0.32 |
| **Weighted Unifrac** |  |  |
| Month | 0.82 | 0.001 |
| Location | 0 | 0.5 |
| Season | 0.63 | 0.001 |
| Sector | 0.01 | 0.16 |
