# Supplementary material for: Spatial-Temporal Survey and Occupancy-Abundance Modeling To Predict Bacterial Community Dynamics in the Drinking Water Microbiome
Source: mBio. 2014 May 27;5(3):e01135-14. doi: 10.1128/mBio.01135-14 (PMC4045074; doi:10.1128/mBio.01135-14)
Supplement: Table S4 — MIC statistics used to construct the network visualization (A) and taxonomy (B) of OTUs selected for this purpose. [file mbo003141850st4.docx]

**Supplementary Table S4. (A)** MIC statistics used to construct the network visualisation and (**B**) taxonomy of OTUs selected for this purpose.

**Table S4A.**

| **Taxa 1** | **Taxa 2** | **MIC (strength)** | **Linear regression (p)** |
| --- | --- | --- | --- |
| Otu0004 | Otu0001 | 0.8494588 | 0.651930592 |
| Otu0007 | Otu0001 | 0.7884406 | -0.678883965 |
| Otu0012 | Otu0001 | 0.807136 | -0.612291311 |
| Otu0012 | Otu0004 | 0.8188634 | -0.662443314 |
| Otu0030 | Otu0008 | 0.7233192 | 0.632189197 |
| Otu0006 | Otu0001 | 0.694046 | -0.53195023 |
| Otu0006 | Otu0004 | 0.734277 | -0.594438612 |
| Otu0012 | Otu0007 | 0.6704899 | 0.633055005 |
| Otu0007 | Otu0004 | 0.7180045 | -0.69535258 |
| Otu0008 | Otu0006 | 0.6890103 | 0.262140492 |
| Otu0058 | Otu0004 | 0.6413101 | -0.31120521 |
| Otu0008 | Otu0001 | 0.6245261 | -0.617292957 |
| Otu0058 | Otu0001 | 0.676581 | -0.283225593 |
| Otu0030 | Otu0006 | 0.6911016 | 0.312853027 |
| Otu0041 | Otu0006 | 0.6655223 | 0.372435695 |
| Otu0058 | Otu0006 | 0.583677 | 0.215799276 |
| Otu0058 | Otu0007 | 0.5857988 | 0.410417277 |
| Otu0030 | Otu0007 | 0.600655 | 0.703172295 |
| Otu0028 | Otu0006 | 0.6456296 | 0.354679232 |
| Otu0012 | Otu0006 | 0.6473991 | 0.507189828 |
| Otu0041 | Otu0001 | 0.6159889 | -0.41180911 |
| Otu0028 | Otu0008 | 0.5784067 | 0.391648333 |
| Otu0014 | Otu0001 | 0.5959048 | -0.574953281 |
| Otu0041 | Otu0004 | 0.6393932 | -0.475376899 |
| Otu0054 | Otu0006 | 0.636807 | 0.669887943 |
| Otu0012 | Otu0008 | 0.5640938 | 0.56000845 |
| Otu0017 | Otu0006 | 0.5662701 | 0.821069753 |
| Otu0093 | Otu0001 | 0.5608891 | -0.470700899 |
| Otu0058 | Otu0012 | 0.5695649 | 0.28285625 |
| Otu0007 | Otu0006 | 0.58052 | 0.417853507 |
| Otu0008 | Otu0007 | 0.5562204 | 0.673756061 |
| Otu0093 | Otu0004 | 0.513357 | -0.493127607 |
| Otu0059 | Otu0001 | 0.5278428 | 0.444221903 |
| Otu0008 | Otu0004 | 0.5679839 | -0.605156166 |
| Otu0030 | Otu0001 | 0.6262381 | -0.525797978 |
| Otu0028 | Otu0001 | 0.5696497 | -0.45047981 |
| Otu0041 | Otu0012 | 0.5803604 | 0.431588511 |
| Otu0014 | Otu0004 | 0.5749684 | -0.605551766 |
| Otu0035 | Otu0001 | 0.562196 | 0.45450986 |
| Otu0054 | Otu0004 | 0.6044851 | -0.575742785 |
| Otu0028 | Otu0004 | 0.5876636 | -0.523393005 |
| Otu0030 | Otu0028 | 0.5648422 | 0.657201959 |
| Otu0023 | Otu0004 | 0.5036342 | 0.325446966 |
| Otu0035 | Otu0004 | 0.5369637 | 0.402703974 |
| Otu0030 | Otu0004 | 0.5875152 | -0.530770662 |
| Otu0016 | Otu0015 | 0.5041906 | 0.735892868 |
| Otu0006 | Otu0002 | 0.5262559 | 0.460927563 |
| Otu0031 | Otu0001 | 0.523121 | -0.545970247 |
| Otu0032 | Otu0006 | 0.5254651 | -0.439117179 |
| Otu0004 | Otu0002 | 0.4888118 | -0.561762071 |
| Otu0020 | Otu0004 | 0.5232696 | -0.572170678 |
| Otu0031 | Otu0004 | 0.5331459 | -0.532649177 |
| Otu0030 | Otu0012 | 0.5323591 | 0.5731921 |
| Otu0023 | Otu0001 | 0.5030589 | 0.428464419 |
| Otu0054 | Otu0012 | 0.5860214 | 0.559961771 |
| Otu0020 | Otu0001 | 0.5329395 | -0.528969258 |
| Otu0028 | Otu0012 | 0.4977308 | 0.485229494 |
| Otu0054 | Otu0001 | 0.5745094 | -0.508624817 |
| Otu0014 | Otu0012 | 0.5286793 | 0.573464796 |
| Otu0008 | Otu0002 | 0.5013825 | 0.470206506 |
| Otu0020 | Otu0012 | 0.5003029 | 0.660201683 |
| Otu0059 | Otu0004 | 0.530102222 | 0.482718317 |
| Otu0028 | Otu0002 | 0.533462121 | 0.386618846 |
| Otu0066 | Otu0001 | 0.495051515 | 0.235735595 |
| Otu0093 | Otu0012 | 0.506070505 | 0.496715659 |
| Otu0053 | Otu0006 | 0.522437273 | -0.451809209 |
| Otu0023 | Otu0007 | 0.495742525 | -0.428765653 |
| Otu0020 | Otu0007 | 0.501224444 | 0.64988697 |
| Otu0061 | Otu0004 | 0.526694545 | 0.633992468 |
| Otu0002 | Otu0001 | 0.469168788 | -0.6056342 |
| Otu0014 | Otu0007 | 0.486831818 | 0.471972163 |
| Otu0032 | Otu0004 | 0.495813838 | 0.444391983 |
| Otu0093 | Otu0006 | 0.518123061 | 0.272632873 |
| Otu0041 | Otu0007 | 0.520929184 | 0.671540571 |
| Otu0061 | Otu0001 | 0.485999898 | 0.356047643 |
| Otu0041 | Otu0008 | 0.489614184 | 0.332690567 |
| Otu0014 | Otu0006 | 0.481340714 | 0.459392779 |
| Otu0061 | Otu0006 | 0.485570928 | -0.357268525 |
| Otu0058 | Otu0008 | 0.48902701 | 0.22940537 |
| Otu0058 | Otu0014 | 0.480196907 | 0.151533527 |
| Otu0028 | Otu0007 | 0.467873918 | 0.650647436 |
| Otu0066 | Otu0004 | 0.487596979 | 0.415897226 |
| Otu0078 | Otu0006 | 0.51745625 | 0.493192527 |
| Otu0025 | Otu0003 | 0.494238958 | -0.364182609 |
| Otu0031 | Otu0012 | 0.480233021 | 0.418607196 |
| Otu0093 | Otu0007 | 0.505792421 | 0.493347001 |
| Otu0019 | Otu0004 | 0.489820526 | 0.345008491 |
| Otu0040 | Otu0004 | 0.481237021 | 0.246642549 |
| Otu0054 | Otu0007 | 0.46195234 | 0.528556289 |
| Otu0054 | Otu0017 | 0.449132872 | 0.672866176 |
| Otu0078 | Otu0054 | 0.47798129 | 0.634962234 |
| Otu0014 | Otu0008 | 0.46729 | 0.389638343 |
| Otu0027 | Otu0001 | 0.474116882 | 0.088545033 |
| Otu0035 | Otu0006 | 0.475495591 | -0.447297726 |
| Otu0035 | Otu0012 | 0.477701087 | -0.445480884 |
| Otu0030 | Otu0002 | 0.470351522 | 0.426207403 |
| Otu0054 | Otu0041 | 0.473698352 | 0.572856232 |
| Otu0035 | Otu0007 | 0.470366067 | -0.493363474 |
| Otu0058 | Otu0041 | 0.470645843 | 0.408393273 |
| Otu0090 | Otu0004 | 0.464072584 | 0.533888421 |
| Otu0024 | Otu0016 | 0.446441461 | 0.789436281 |
| Otu0090 | Otu0006 | 0.462506404 | -0.331572093 |
| Otu0044 | Otu0004 | 0.462681124 | 0.40060058 |
| Otu0059 | Otu0007 | 0.469746092 | -0.429561483 |
| Otu0012 | Otu0002 | 0.450727126 | 0.580272313 |
| Otu0099 | Otu0001 | 0.488728953 | -0.466061968 |
| Otu0034 | Otu0004 | 0.461578488 | 0.561970027 |
| Otu0025 | Otu0009 | 0.447134302 | 0.486353565 |
| Otu0020 | Otu0006 | 0.434675116 | 0.341728998 |
| Otu0041 | Otu0030 | 0.46530907 | 0.627350509 |
| Otu0059 | Otu0006 | 0.479227647 | -0.378967121 |
| Otu0041 | Otu0028 | 0.453467647 | 0.822110239 |
| Otu0044 | Otu0001 | 0.468037711 | 0.382856435 |
| Otu0014 | Otu0002 | 0.453093735 | 0.51977058 |
| Otu0053 | Otu0004 | 0.465631098 | 0.501535825 |
| Otu0053 | Otu0001 | 0.458947927 | 0.426414387 |
| Otu0066 | Otu0006 | 0.458048101 | -0.411309557 |
| Otu0023 | Otu0012 | 0.446417532 | -0.323560853 |
| Otu0031 | Otu0007 | 0.450426447 | 0.616800911 |
| Otu0031 | Otu0006 | 0.4466704 | 0.413850909 |
| Otu0078 | Otu0004 | 0.465980405 | -0.49520311 |
| Otu0061 | Otu0012 | 0.446769178 | -0.382642242 |
| Otu0066 | Otu0007 | 0.463542083 | -0.450526263 |
| Otu0078 | Otu0017 | 0.442220986 | 0.426405857 |
| Otu0093 | Otu0008 | 0.460843429 | 0.406824115 |
| Otu0019 | Otu0006 | 0.442859857 | -0.443907529 |
| Otu0099 | Otu0004 | 0.465195143 | -0.470659546 |
| Otu0034 | Otu0006 | 0.45205971 | -0.257413923 |
| Otu0041 | Otu0002 | 0.447435147 | 0.286344195 |
| Otu0023 | Otu0006 | 0.440872941 | -0.317252227 |
| Otu0058 | Otu0030 | 0.449497647 | 0.301080566 |
| Otu0023 | Otu0002 | 0.445985821 | -0.456116445 |
| Otu0053 | Otu0008 | 0.460634776 | -0.497450905 |
| Otu0032 | Otu0012 | 0.43808403 | -0.432427832 |
| Otu0019 | Otu0001 | 0.447353731 | 0.44630557 |
| Otu0032 | Otu0001 | 0.440558333 | 0.344682828 |
| Otu0030 | Otu0014 | 0.441637121 | 0.260813112 |
| Otu0067 | Otu0006 | 0.441754 | -0.316040024 |
| Otu0054 | Otu0002 | 0.452525692 | 0.444264862 |
| Otu0099 | Otu0007 | 0.459695231 | 0.608086139 |
| Otu0090 | Otu0001 | 0.438018438 | 0.256291546 |
| Otu0053 | Otu0012 | 0.449280469 | -0.453014084 |
| Otu0024 | Otu0015 | 0.424342222 | 0.646502504 |
| Otu0054 | Otu0028 | 0.443368571 | 0.584312451 |
| Otu0058 | Otu0031 | 0.438908889 | 0.283228113 |
| Otu0092 | Otu0004 | 0.447284194 | 0.454276613 |
| Otu0059 | Otu0012 | 0.44535459 | -0.373743956 |
| Otu0032 | Otu0002 | 0.440186557 | -0.345264895 |
| Otu0027 | Otu0004 | 0.443859836 | 0.345402499 |
| Otu0019 | Otu0012 | 0.450238033 | -0.45171276 |
| Otu0100 | Otu0001 | 0.451613051 | 0.399092495 |
| Otu0041 | Otu0020 | 0.430615254 | 0.544906606 |
| Otu0040 | Otu0001 | 0.446961017 | 0.16237217 |
| Otu0034 | Otu0001 | 0.435500877 | 0.323990794 |
| Otu0053 | Otu0007 | 0.445504821 | -0.483340743 |
| Otu0058 | Otu0028 | 0.446672143 | 0.401404283 |
| Otu0058 | Otu0054 | 0.440476415 | 0.26910392 |
| Otu0078 | Otu0001 | 0.437570588 | -0.41330637 |
| Otu0067 | Otu0001 | 0.447645098 | 0.142898455 |
| Otu0071 | Otu0016 | 0.426996078 | 0.65044097 |
| Otu0018 | Otu0008 | 0.439829608 | 0.55269392 |
| Otu0030 | Otu0023 | 0.430397 | -0.344842931 |
| Otu0092 | Otu0001 | 0.435163 | 0.271330136 |
| Otu0066 | Otu0012 | 0.440866122 | -0.433676017 |
| Otu0054 | Otu0032 | 0.432706327 | -0.398896863 |
| Otu0092 | Otu0006 | 0.445554286 | -0.349368983 |
| Otu0046 | Otu0004 | 0.437118125 | 0.53741184 |
| Otu0093 | Otu0020 | 0.436724375 | 0.519965651 |
| Otu0028 | Otu0020 | 0.424352917 | 0.541376147 |
| Otu0099 | Otu0012 | 0.445013125 | 0.516661253 |
| Otu0044 | Otu0006 | 0.435990625 | -0.442983861 |
| Otu0100 | Otu0006 | 0.438806383 | -0.320682873 |
| Otu0029 | Otu0008 | 0.434516383 | 0.59526691 |
| Otu0054 | Otu0019 | 0.431960222 | -0.418177169 |
| Otu0085 | Otu0004 | 0.442043778 | 0.180074144 |
| Otu0054 | Otu0014 | 0.435104 | 0.496396823 |
| Otu0053 | Otu0002 | 0.434313333 | -0.427418228 |
| Otu0013 | Otu0001 | 0.433306364 | -0.550437015 |
| Otu0053 | Otu0030 | 0.4419975 | -0.44394491 |
| Otu0093 | Otu0030 | 0.441588837 | 0.390019955 |
| Otu0093 | Otu0028 | 0.434456429 | 0.45496607 |
| Otu0028 | Otu0014 | 0.436935476 | 0.222052031 |
| Otu0077 | Otu0007 | 0.43997 | 0.577702242 |
| Otu0067 | Otu0015 | 0.4312 | 0.62026478 |
| Otu0078 | Otu0012 | 0.448605854 | 0.487189265 |
| Otu0112 | Otu0004 | 0.439202195 | 0.350485239 |
| Otu0112 | Otu0001 | 0.439011707 | 0.316481548 |
| Otu0007 | Otu0003 | 0.428708205 | 0.387160195 |
| Otu0023 | Otu0014 | 0.435836842 | -0.348623291 |
| Otu0142 | Otu0001 | 0.442421842 | 0.317522852 |
| Otu0019 | Otu0007 | 0.423955135 | -0.427169642 |
| Otu0023 | Otu0008 | 0.425655 | -0.370405814 |
| Otu0045 | Otu0006 | 0.451542222 | -0.369338636 |
| Otu0054 | Otu0035 | 0.431651944 | -0.427496614 |
| Otu0006 | Otu0005 | 0.419001111 | -0.431236103 |
| Otu0061 | Otu0007 | 0.446435429 | -0.389494968 |
| Otu0031 | Otu0008 | 0.433969412 | 0.446469259 |
| Otu0009 | Otu0006 | 0.422132121 | 0.418367908 |
| Otu0077 | Otu0001 | 0.428821875 | -0.45490579 |
| Otu0020 | Otu0008 | 0.422240625 | 0.592784149 |
| Otu0031 | Otu0014 | 0.42676625 | 0.402857393 |
| Otu0093 | Otu0013 | 0.425645625 | 0.569843785 |
| Otu0077 | Otu0004 | 0.440413871 | -0.429125785 |
| Otu0100 | Otu0004 | 0.430468667 | 0.445452722 |
| Otu0106 | Otu0006 | 0.435558276 | 0.601459792 |
| Otu0072 | Otu0008 | 0.428743103 | 0.540406837 |
| Otu0089 | Otu0004 | 0.42936069 | 0.609957555 |
| Otu0142 | Otu0004 | 0.429773793 | 0.363860141 |
| Otu0084 | Otu0007 | 0.433370357 | -0.425332453 |
| Otu0099 | Otu0008 | 0.424531786 | 0.500946099 |
| Otu0065 | Otu0006 | 0.41992 | -0.31310626 |
| Otu0078 | Otu0005 | 0.4262836 | -0.356515386 |
| Otu0054 | Otu0008 | 0.4233656 | 0.267852106 |
| Otu0041 | Otu0014 | 0.4284224 | 0.132010702 |
| Otu0092 | Otu0008 | 0.42246125 | -0.390368468 |
| Otu0005 | Otu0004 | 0.418443913 | 0.398090263 |
| Otu0053 | Otu0014 | 0.432484348 | -0.395575161 |
| Otu0125 | Otu0001 | 0.426627391 | 0.345930326 |
| Otu0067 | Otu0004 | 0.430206522 | 0.236262318 |
| Otu0030 | Otu0020 | 0.424351739 | 0.594043443 |
| Otu0079 | Otu0001 | 0.430803043 | 0.356204613 |
| Otu0031 | Otu0030 | 0.435452174 | 0.457585446 |
| Otu0077 | Otu0008 | 0.430705217 | 0.503426368 |
| Otu0017 | Otu0009 | 0.412164545 | 0.493868945 |
| Otu0054 | Otu0030 | 0.43001 | 0.467658461 |
| Otu0015 | Otu0006 | 0.427055455 | -0.328546406 |
| Otu0058 | Otu0002 | 0.430024762 | 0.16901534 |
| Otu0079 | Otu0007 | 0.431334286 | -0.482514722 |
| Otu0143 | Otu0004 | 0.4251255 | 0.372009647 |
| Otu0007 | Otu0002 | 0.4203525 | 0.418665102 |
| Otu0016 | Otu0009 | 0.424713 | 0.512434909 |
| Otu0066 | Otu0008 | 0.436380526 | -0.452018993 |
| Otu0053 | Otu0028 | 0.432394211 | -0.374307764 |
| Otu0026 | Otu0006 | 0.427404737 | 0.178601929 |
| Otu0054 | Otu0005 | 0.414863684 | -0.39783689 |
| Otu0099 | Otu0006 | 0.426670556 | 0.230707207 |
| Otu0085 | Otu0001 | 0.426845 | 0.173858777 |
| Otu0041 | Otu0017 | 0.426606667 | 0.363048553 |
| Otu0071 | Otu0015 | 0.414109412 | 0.693808849 |
| Otu0084 | Otu0001 | 0.434673125 | 0.403526824 |
| Otu0018 | Otu0006 | 0.419164375 | 0.014318126 |
| Otu0062 | Otu0006 | 0.420971875 | -0.393152309 |
| Otu0034 | Otu0009 | 0.432603125 | -0.236360439 |
| Otu0041 | Otu0035 | 0.423583333 | -0.308738846 |
| Otu0029 | Otu0007 | 0.414216 | 0.559397949 |
| Otu0059 | Otu0002 | 0.425147333 | -0.411900409 |
| Otu0041 | Otu0032 | 0.419412857 | -0.316378036 |
| Otu0056 | Otu0009 | 0.426878571 | -0.353651204 |
| Otu0013 | Otu0008 | 0.414842857 | 0.469859085 |
| Otu0093 | Otu0041 | 0.42456 | 0.387313194 |
| Otu0058 | Otu0020 | 0.42184 | 0.290336766 |
| Otu0044 | Otu0012 | 0.426022857 | -0.439357301 |
| Otu0090 | Otu0008 | 0.420346154 | -0.370000008 |
| Otu0084 | Otu0003 | 0.423700769 | -0.399768567 |
| Otu0071 | Otu0001 | 0.418740769 | 0.023424763 |
| Otu0003 | Otu0001 | 0.41721 | -0.455621981 |
| Otu0054 | Otu0020 | 0.4123325 | 0.476218414 |
| Otu0028 | Otu0018 | 0.41556 | 0.190784598 |
| Otu0057 | Otu0006 | 0.4290775 | -0.366587063 |
| Otu0061 | Otu0002 | 0.416805 | -0.331549825 |
| Otu0112 | Otu0006 | 0.422567273 | -0.350287345 |
| Otu0029 | Otu0001 | 0.425644545 | -0.493909426 |
| Otu0065 | Otu0004 | 0.409633636 | 0.3162673 |
| Otu0100 | Otu0008 | 0.420954545 | -0.386979894 |
| Otu0061 | Otu0041 | 0.415527273 | -0.262250833 |
| Otu0034 | Otu0008 | 0.42885 | -0.325435238 |
| Otu0058 | Otu0023 | 0.423206364 | -0.154229861 |
| Otu0125 | Otu0006 | 0.423194 | -0.328796327 |
| Otu0032 | Otu0028 | 0.431545 | -0.362072142 |
| Otu0059 | Otu0014 | 0.418162 | -0.3809015 |
| Otu0093 | Otu0014 | 0.429247 | 0.360164339 |
| Otu0047 | Otu0001 | 0.443983 | 0.085869868 |
| Otu0044 | Otu0007 | 0.427398 | -0.444562765 |
| Otu0066 | Otu0030 | 0.415193 | -0.416779269 |
| Otu0061 | Otu0014 | 0.420851 | -0.373604298 |
| Otu0090 | Otu0059 | 0.445051 | 0.504913485 |
| Otu0079 | Otu0004 | 0.442179 | 0.414240557 |
| Otu0034 | Otu0002 | 0.424734 | -0.249188415 |
| Otu0022 | Otu0001 | 0.433733 | 0.16430874 |
| Otu0078 | Otu0007 | 0.426791 | 0.430036966 |
| Otu0018 | Otu0001 | 0.42916 | -0.423732644 |
| Otu0023 | Otu0003 | 0.430631 | -0.364401924 |
| Otu0059 | Otu0035 | 0.414833 | 0.568942732 |
| Otu0118 | No significant association |  |  |
| Otu0117 | No significant association |  |  |
| Otu0116 | No significant association |  |  |
| Otu0115 | No significant association |  |  |
| Otu0114 | No significant association |  |  |
| Otu0111 | No significant association |  |  |
| Otu0109 | No significant association |  |  |
| Otu0108 | No significant association |  |  |
| Otu0107 | No significant association |  |  |
| Otu0105 | No significant association |  |  |
| Otu0104 | No significant association |  |  |
| Otu0103 | No significant association |  |  |
| Otu0102 | No significant association |  |  |
| Otu0101 | No significant association |  |  |
| Otu0098 | No significant association |  |  |
| Otu0097 | No significant association |  |  |
| Otu0096 | No significant association |  |  |
| Otu0095 | No significant association |  |  |
| Otu0094 | No significant association |  |  |
| Otu0091 | No significant association |  |  |
| Otu0088 | No significant association |  |  |
| Otu0087 | No significant association |  |  |
| Otu0086 | No significant association |  |  |
| Otu0083 | No significant association |  |  |
| Otu0082 | No significant association |  |  |
| Otu0081 | No significant association |  |  |
| Otu0080 | No significant association |  |  |
| Otu0076 | No significant association |  |  |
| Otu0075 | No significant association |  |  |
| Otu0074 | No significant association |  |  |
| Otu0073 | No significant association |  |  |
| Otu0070 | No significant association |  |  |
| Otu0069 | No significant association |  |  |
| Otu0068 | No significant association |  |  |
| Otu0064 | No significant association |  |  |
| Otu0063 | No significant association |  |  |
| Otu0060 | No significant association |  |  |
| Otu0055 | No significant association |  |  |
| Otu0052 | No significant association |  |  |
| Otu0050 | No significant association |  |  |
| Otu0049 | No significant association |  |  |
| Otu0048 | No significant association |  |  |
| Otu0043 | No significant association |  |  |
| Otu0042 | No significant association |  |  |
| Otu0039 | No significant association |  |  |
| Otu0038 | No significant association |  |  |
| Otu0037 | No significant association |  |  |
| Otu0036 | No significant association |  |  |
| Otu0033 | No significant association |  |  |
| Otu0021 | No significant association |  |  |
| Otu0011 | No significant association |  |  |
| Otu0010 | No significant association |  |  |
| Otu0119 | No significant association |  |  |
| Otu0121 | No significant association |  |  |
| Otu0122 | No significant association |  |  |
| Otu0123 | No significant association |  |  |
| Otu0124 | No significant association |  |  |
| Otu0128 | No significant association |  |  |
| Otu0131 | No significant association |  |  |
| Otu0133 | No significant association |  |  |
| Otu0134 | No significant association |  |  |
| Otu0135 | No significant association |  |  |
| Otu0136 | No significant association |  |  |
| Otu0137 | No significant association |  |  |
| Otu0138 | No significant association |  |  |
| Otu0139 | No significant association |  |  |
| Otu0144 | No significant association |  |  |
| Otu0145 | No significant association |  |  |
| Otu0146 | No significant association |  |  |
| Otu0148 | No significant association |  |  |
| Otu0150 | No significant association |  |  |
| Otu0155 | No significant association |  |  |
| Otu0157 | No significant association |  |  |
| Otu0159 | No significant association |  |  |
| Otu0163 | No significant association |  |  |
| Otu0164 | No significant association |  |  |
| Otu0169 | No significant association |  |  |
| Otu0171 | No significant association |  |  |
| Otu0180 | No significant association |  |  |

**Table S4B.**

| **OTU #** | **Taxonomy** |
| --- | --- |
| 0001 | Bacteria(100);"Proteobacteria"(100);Betaproteobacteria(100);Burkholderiales(100);Comamonadaceae(100);Acidovorax(93); |
| 0002 | Bacteria(100);"Proteobacteria"(100);Betaproteobacteria(100);Burkholderiales(100);Comamonadaceae(100);Hydrogenophaga(98); |
| 0003 | Bacteria(100);"Proteobacteria"(100);Alphaproteobacteria(100);Caulobacterales(100);Caulobacteraceae(100);Brevundimonas(100); |
| 0004 | Bacteria(100);"Proteobacteria"(100);Betaproteobacteria(100);Rhodocyclales(100);Rhodocyclaceae(100);Georgfuchsia(98); |
| 0005 | Bacteria(100);OD1(100);OD1_class_incertae_sedis(100);OD1_order_incertae_sedis(100);OD1_family_incertae_sedis(100);OD1_genus_incertae_sedis(100); |
| 0006 | Bacteria(100);"Proteobacteria"(100);Gammaproteobacteria(100);Pseudomonadales(100);Pseudomonadaceae(100);Pseudomonas(100); |
| 0007 | Bacteria(100);"Proteobacteria"(76);unclassified(76);unclassified(76);unclassified(76);unclassified(76); |
| 0008 | Bacteria(100);"Proteobacteria"(98);unclassified(98);unclassified(98);unclassified(98);unclassified(98); |
| 0009 | Bacteria(100);unclassified(100);unclassified(100);unclassified(100);unclassified(100);unclassified(100); |
| 0010 | Bacteria(100);"Proteobacteria"(100);Alphaproteobacteria(100);Rhizobiales(100);Bradyrhizobiaceae(100);Bosea(100); |
| 0011 | Bacteria(100);"Proteobacteria"(100);Betaproteobacteria(100);unclassified(100);unclassified(100);unclassified(100); |
| 0012 | Bacteria(100);"Proteobacteria"(100);Alphaproteobacteria(100);Sphingomonadales(100);Sphingomonadaceae(99);unclassified(99); |
| 0013 | Bacteria(100);unclassified(88);unclassified(88);unclassified(88);unclassified(88);unclassified(88); |
| 0014 | Bacteria(100);"Proteobacteria"(100);Alphaproteobacteria(100);Rhodobacterales(100);Rhodobacteraceae(100);Rhodobacter(61); |
| 0015 | Bacteria(100);unclassified(100);unclassified(100);unclassified(100);unclassified(100);unclassified(100); |
| 0016 | Bacteria(100);unclassified(100);unclassified(100);unclassified(100);unclassified(100);unclassified(100); |
| 0017 | Bacteria(100);"Proteobacteria"(100);Betaproteobacteria(100);Burkholderiales(100);Oxalobacteraceae(100);Herminiimonas(96); |
| 0018 | Bacteria(100);"Proteobacteria"(100);unclassified(100);unclassified(100);unclassified(100);unclassified(100); |
| 0019 | Bacteria(100);"Proteobacteria"(100);Alphaproteobacteria(100);Rhodospirillales(100);Acetobacteraceae(100);unclassified(100); |
| 0020 | Bacteria(100);"Proteobacteria"(100);Betaproteobacteria(100);Nitrosomonadales(88);Nitrosomonadaceae(88);unclassified(88); |
| 0021 | Bacteria(100);unclassified(100);unclassified(100);unclassified(100);unclassified(100);unclassified(100); |
| 0022 | Bacteria(100);"Proteobacteria"(100);Betaproteobacteria(100);Burkholderiales(100);unclassified(99);unclassified(99); |
| 0023 | Bacteria(100);OD1(100);OD1_class_incertae_sedis(100);OD1_order_incertae_sedis(100);OD1_family_incertae_sedis(100);OD1_genus_incertae_sedis(100); |
| 0024 | Bacteria(100);OD1(100);OD1_class_incertae_sedis(100);OD1_order_incertae_sedis(100);OD1_family_incertae_sedis(100);OD1_genus_incertae_sedis(100); |
| 0025 | Bacteria(100);"Proteobacteria"(100);Betaproteobacteria(100);Methylophilales(100);Methylophilaceae(100);Methylophilus(100); |
| 0026 | Bacteria(100);"Proteobacteria"(100);Gammaproteobacteria(100);Legionellales(96);Legionellaceae(96);Legionella(96); |
| 0027 | Bacteria(100);"Proteobacteria"(100);Deltaproteobacteria(100);Bdellovibrionales(100);Bdellovibrionaceae(100);Bdellovibrio(100); |
| 0028 | Bacteria(100);unclassified(100);unclassified(100);unclassified(100);unclassified(100);unclassified(100); |
| 0029 | Bacteria(100);unclassified(100);unclassified(100);unclassified(100);unclassified(100);unclassified(100); |
| 0030 | Bacteria(100);"Proteobacteria"(100);unclassified(100);unclassified(100);unclassified(100);unclassified(100); |
| 0031 | Bacteria(100);unclassified(100);unclassified(100);unclassified(100);unclassified(100);unclassified(100); |
| 0032 | Bacteria(100);"Proteobacteria"(100);Alphaproteobacteria(100);Rhizobiales(100);unclassified(99);unclassified(99); |
| 0033 | Bacteria(100);"Proteobacteria"(100);Betaproteobacteria(100);Burkholderiales(100);Comamonadaceae(100);Hydrogenophaga(100); |
| 0034 | Bacteria(100);"Acidobacteria"(100);Acidobacteria_Gp4(100);Acidobacteria_Gp4_order_incertae_sedis(100);Acidobacteria_Gp4_family_incertae_sedis(100);Gp4(100); |
| 0035 | Bacteria(100);unclassified(100);unclassified(100);unclassified(100);unclassified(100);unclassified(100); |
| 0036 | Bacteria(100);"Proteobacteria"(100);Alphaproteobacteria(100);Caulobacterales(100);Caulobacteraceae(100);unclassified(98); |
| 0037 | Bacteria(100);"Proteobacteria"(100);Alphaproteobacteria(100);Rhodospirillales(100);Acetobacteraceae(100);Roseomonas(100); |
| 0038 | Bacteria(100);unclassified(100);unclassified(100);unclassified(100);unclassified(100);unclassified(100); |
| 0039 | Bacteria(100);"Proteobacteria"(100);Deltaproteobacteria(100);Bdellovibrionales(100);Bdellovibrionaceae(100);Bdellovibrio(100); |
| 0040 | Bacteria(100);"Proteobacteria"(100);Deltaproteobacteria(100);unclassified(100);unclassified(100);unclassified(100); |
| 0041 | Bacteria(100);"Proteobacteria"(100);unclassified(100);unclassified(100);unclassified(100);unclassified(100); |
| 0042 | Bacteria(100);"Proteobacteria"(100);Alphaproteobacteria(100);Rhodospirillales(100);Acetobacteraceae(100);unclassified(100); |
| 0043 | Bacteria(100);"Proteobacteria"(100);Deltaproteobacteria(100);Bdellovibrionales(100);Bdellovibrionaceae(100);Bdellovibrio(100); |
| 0044 | Bacteria(100);"Proteobacteria"(100);Betaproteobacteria(100);unclassified(100);unclassified(100);unclassified(100); |
| 0045 | Bacteria(100);"Proteobacteria"(100);Alphaproteobacteria(100);Rhodospirillales(100);Acetobacteraceae(100);Roseococcus(100); |
| 0046 | Bacteria(100);"Proteobacteria"(98);unclassified(94);unclassified(94);unclassified(94);unclassified(94); |
| 0047 | Bacteria(100);OD1(100);OD1_class_incertae_sedis(100);OD1_order_incertae_sedis(100);OD1_family_incertae_sedis(100);OD1_genus_incertae_sedis(100); |
| 0048 | Bacteria(100);"Proteobacteria"(100);unclassified(72);unclassified(72);unclassified(72);unclassified(72); |
| 0049 | Bacteria(100);"Proteobacteria"(100);Betaproteobacteria(100);unclassified(97);unclassified(97);unclassified(97); |
| 0050 | Bacteria(100);unclassified(100);unclassified(100);unclassified(100);unclassified(100);unclassified(100); |
| 0052 | Bacteria(100);"Proteobacteria"(100);Betaproteobacteria(100);unclassified(78);unclassified(78);unclassified(78); |
| 0053 | Bacteria(100);"Proteobacteria"(100);Alphaproteobacteria(100);Rhodospirillales(100);Acetobacteraceae(100);unclassified(100); |
| 0054 | Bacteria(100);"Proteobacteria"(100);Alphaproteobacteria(100);Sphingomonadales(100);unclassified(100);unclassified(100); |
| 0055 | Bacteria(100);OD1(100);OD1_class_incertae_sedis(100);OD1_order_incertae_sedis(100);OD1_family_incertae_sedis(100);OD1_genus_incertae_sedis(100); |
| 0056 | Bacteria(100);"Proteobacteria"(100);Alphaproteobacteria(100);Rhizobiales(100);Hyphomicrobiaceae(100);Hyphomicrobium(100); |
| 0057 | Bacteria(100);"Proteobacteria"(100);Gammaproteobacteria(100);Xanthomonadales(100);Xanthomonadaceae(100);unclassified(99); |
| 0058 | Bacteria(100);"Proteobacteria"(100);unclassified(100);unclassified(100);unclassified(100);unclassified(100); |
| 0059 | Bacteria(100);"Proteobacteria"(100);Deltaproteobacteria(100);Bdellovibrionales(100);Bdellovibrionaceae(100);Bdellovibrio(100); |
| 0060 | Bacteria(100);"Proteobacteria"(100);Betaproteobacteria(100);Burkholderiales(100);unclassified(92);unclassified(92); |
| 0061 | Bacteria(100);"Proteobacteria"(100);Betaproteobacteria(100);Burkholderiales(100);Burkholderiaceae(100);Limnobacter(100); |
| 0062 | Bacteria(100);OD1(100);OD1_class_incertae_sedis(100);OD1_order_incertae_sedis(100);OD1_family_incertae_sedis(100);OD1_genus_incertae_sedis(100); |
| 0063 | Bacteria(100);"Proteobacteria"(100);Betaproteobacteria(100);unclassified(100);unclassified(100);unclassified(100); |
| 0064 | Bacteria(100);"Proteobacteria"(100);Alphaproteobacteria(100);Rhizobiales(100);Methylobacteriaceae(100);Methylobacterium(100); |
| 0065 | Bacteria(100);"Gemmatimonadetes"(100);Gemmatimonadetes(100);Gemmatimonadales(100);Gemmatimonadaceae(100);Gemmatimonas(100); |
| 0066 | Bacteria(100);"Proteobacteria"(100);Betaproteobacteria(100);unclassified(84);unclassified(84);unclassified(84); |
| 0067 | Bacteria(100);"Proteobacteria"(100);Gammaproteobacteria(100);Xanthomonadales(100);Xanthomonadaceae(100);unclassified(99); |
| 0068 | Bacteria(100);"Proteobacteria"(100);Deltaproteobacteria(93);Bdellovibrionales(79);Bdellovibrionaceae(79);Bdellovibrio(79); |
| 0069 | Bacteria(100);"Proteobacteria"(100);Gammaproteobacteria(100);Legionellales(90);Legionellaceae(90);Legionella(90); |
| 0070 | Bacteria(100);"Proteobacteria"(100);unclassified(100);unclassified(100);unclassified(100);unclassified(100); |
| 0071 | Bacteria(100);"Proteobacteria"(100);unclassified(100);unclassified(100);unclassified(100);unclassified(100); |
| 0072 | Bacteria(100);"Proteobacteria"(100);unclassified(100);unclassified(100);unclassified(100);unclassified(100); |
| 0073 | Bacteria(100);"Proteobacteria"(100);Deltaproteobacteria(100);Bdellovibrionales(100);Bdellovibrionaceae(100);Bdellovibrio(100); |
| 0074 | Bacteria(100);"Proteobacteria"(100);Alphaproteobacteria(100);Rhizobiales(100);Hyphomicrobiaceae(100);unclassified(91); |
| 0075 | Bacteria(100);OD1(100);OD1_class_incertae_sedis(100);OD1_order_incertae_sedis(100);OD1_family_incertae_sedis(100);OD1_genus_incertae_sedis(100); |
| 0076 | Bacteria(100);"Proteobacteria"(100);Betaproteobacteria(100);unclassified(100);unclassified(100);unclassified(100); |
| 0077 | Bacteria(100);"Proteobacteria"(100);Betaproteobacteria(100);Burkholderiales(100);Oxalobacteraceae(89);unclassified(82); |
| 0078 | Bacteria(100);"Proteobacteria"(100);Alphaproteobacteria(100);Sphingomonadales(100);Sphingomonadaceae(100);Sphingomonas(90); |
| 0079 | Bacteria(100);"Proteobacteria"(100);Betaproteobacteria(100);Burkholderiales(100);Comamonadaceae(100);Hydrogenophaga(98); |
| 0080 | Bacteria(100);"Proteobacteria"(100);Deltaproteobacteria(100);Bdellovibrionales(100);Bdellovibrionaceae(100);Bdellovibrio(100); |
| 0081 | Bacteria(100);unclassified(97);unclassified(97);unclassified(97);unclassified(97);unclassified(97); |
| 0082 | Bacteria(100);"Chlamydiae"(100);Chlamydiae(100);Chlamydiales(100);unclassified(98);unclassified(98); |
| 0083 | Bacteria(100);"Proteobacteria"(100);Alphaproteobacteria(100);unclassified(100);unclassified(100);unclassified(100); |
| 0084 | Bacteria(100);unclassified(100);unclassified(100);unclassified(100);unclassified(100);unclassified(100); |
| 0085 | Bacteria(100);"Proteobacteria"(100);Gammaproteobacteria(100);Xanthomonadales(100);Xanthomonadaceae(100);Silanimonas(100); |
| 0086 | Bacteria(100);"Proteobacteria"(100);Deltaproteobacteria(100);Bdellovibrionales(100);Bdellovibrionaceae(100);Bdellovibrio(100); |
| 0087 | Bacteria(100);"Proteobacteria"(100);Gammaproteobacteria(100);unclassified(100);unclassified(100);unclassified(100); |
| 0088 | Bacteria(100);unclassified(100);unclassified(100);unclassified(100);unclassified(100);unclassified(100); |
| 0089 | Bacteria(100);unclassified(100);unclassified(100);unclassified(100);unclassified(100);unclassified(100); |
| 0090 | Bacteria(100);"Proteobacteria"(100);unclassified(100);unclassified(100);unclassified(100);unclassified(100); |
| 0091 | Bacteria(100);unclassified(100);unclassified(100);unclassified(100);unclassified(100);unclassified(100); |
| 0092 | Bacteria(100);"Proteobacteria"(100);Deltaproteobacteria(100);Bdellovibrionales(100);Bdellovibrionaceae(100);Bdellovibrio(100); |
| 0093 | Bacteria(100);unclassified(100);unclassified(100);unclassified(100);unclassified(100);unclassified(100); |
| 0094 | Bacteria(100);"Proteobacteria"(100);Gammaproteobacteria(100);Legionellales(100);Legionellaceae(100);Legionella(100); |
| 0095 | Bacteria(100);"Proteobacteria"(100);Deltaproteobacteria(100);Bdellovibrionales(100);Bdellovibrionaceae(100);Bdellovibrio(100); |
| 0096 | Bacteria(100);"Proteobacteria"(100);Gammaproteobacteria(100);Legionellales(100);Legionellaceae(100);Legionella(100); |
| 0097 | Bacteria(100);"Proteobacteria"(100);Alphaproteobacteria(100);Caulobacterales(100);Hyphomonadaceae(100);unclassified(100); |
| 0098 | Bacteria(100);"Actinobacteria"(100);Actinobacteria(100);Actinomycetales(100);Mycobacteriaceae(100);Mycobacterium(100); |
| 0099 | Bacteria(100);"Chlamydiae"(100);Chlamydiae(100);Chlamydiales(100);Parachlamydiaceae(100);unclassified(97); |
| 0100 | Bacteria(100);"Proteobacteria"(100);Betaproteobacteria(100);unclassified(100);unclassified(100);unclassified(100); |
| 0101 | Bacteria(100);OD1(100);OD1_class_incertae_sedis(100);OD1_order_incertae_sedis(100);OD1_family_incertae_sedis(100);OD1_genus_incertae_sedis(100); |
| 0102 | Bacteria(100);"Nitrospira"(100);"Nitrospira"(100);"Nitrospirales"(100);"Nitrospiraceae"(100);Nitrospira(100); |
| 0103 | Bacteria(100);"Proteobacteria"(100);Alphaproteobacteria(100);Rhodospirillales(96);unclassified(96);unclassified(96); |
| 0104 | Bacteria(100);"Proteobacteria"(100);Gammaproteobacteria(100);Xanthomonadales(100);Xanthomonadaceae(100);unclassified(57); |
| 0105 | Bacteria(100);"Proteobacteria"(98);unclassified(98);unclassified(98);unclassified(98);unclassified(98); |
| 0106 | Bacteria(100);unclassified(100);unclassified(100);unclassified(100);unclassified(100);unclassified(100); |
| 0107 | Bacteria(100);unclassified(100);unclassified(100);unclassified(100);unclassified(100);unclassified(100); |
| 0108 | Bacteria(100);"Proteobacteria"(100);unclassified(51);unclassified(51);unclassified(51);unclassified(51); |
| 0109 | Bacteria(100);unclassified(100);unclassified(100);unclassified(100);unclassified(100);unclassified(100); |
| 0111 | Bacteria(100);"Planctomycetes"(100);"Planctomycetacia"(100);Planctomycetales(100);Planctomycetaceae(100);unclassified(100); |
| 0112 | Bacteria(100);"Proteobacteria"(100);Deltaproteobacteria(100);Bdellovibrionales(100);Bdellovibrionaceae(100);Bdellovibrio(100); |
| 0114 | Bacteria(100);"Proteobacteria"(100);unclassified(100);unclassified(100);unclassified(100);unclassified(100); |
| 0115 | Bacteria(100);"Proteobacteria"(100);Alphaproteobacteria(100);Rhizobiales(100);unclassified(100);unclassified(100); |
| 0116 | Bacteria(100);OD1(100);OD1_class_incertae_sedis(100);OD1_order_incertae_sedis(100);OD1_family_incertae_sedis(100);OD1_genus_incertae_sedis(100); |
| 0117 | Bacteria(100);"Proteobacteria"(100);Alphaproteobacteria(100);Rhizobiales(100);Phyllobacteriaceae(100);Mesorhizobium(94); |
| 0118 | Bacteria(100);unclassified(100);unclassified(100);unclassified(100);unclassified(100);unclassified(100); |
| 0119 | Bacteria(100);"Proteobacteria"(100);Betaproteobacteria(100);Burkholderiales(100);Burkholderiaceae(100);Polynucleobacter(100); |
| 0121 | Bacteria(100);"Proteobacteria"(100);Alphaproteobacteria(100);Rhizobiales(100);Bradyrhizobiaceae(100);Afipia(84); |
| 0122 | Bacteria(100);unclassified(100);unclassified(100);unclassified(100);unclassified(100);unclassified(100); |
| 0123 | Bacteria(100);OD1(100);OD1_class_incertae_sedis(100);OD1_order_incertae_sedis(100);OD1_family_incertae_sedis(100);OD1_genus_incertae_sedis(100); |
| 0124 | Bacteria(100);"Proteobacteria"(100);Alphaproteobacteria(100);unclassified(100);unclassified(100);unclassified(100); |
| 0125 | Bacteria(100);unclassified(100);unclassified(100);unclassified(100);unclassified(100);unclassified(100); |
| 0128 | Bacteria(100);unclassified(100);unclassified(100);unclassified(100);unclassified(100);unclassified(100); |
| 0131 | Bacteria(100);"Proteobacteria"(100);Alphaproteobacteria(100);Rhizobiales(100);Methylocystaceae(84);unclassified(84); |
| 0133 | Bacteria(100);"Proteobacteria"(100);Betaproteobacteria(100);unclassified(100);unclassified(100);unclassified(100); |
| 0134 | Bacteria(100);unclassified(100);unclassified(100);unclassified(100);unclassified(100);unclassified(100); |
| 0135 | Bacteria(100);"Proteobacteria"(99);Deltaproteobacteria(58);unclassified(58);unclassified(58);unclassified(58); |
| 0136 | Bacteria(100);"Acidobacteria"(100);Acidobacteria_Gp3(100);unclassified(99);unclassified(99);unclassified(99); |
| 0137 | Bacteria(100);"Proteobacteria"(100);Alphaproteobacteria(100);Rhodospirillales(100);Acetobacteraceae(100);Roseomonas(100); |
| 0138 | Bacteria(100);"Proteobacteria"(100);Deltaproteobacteria(100);Bdellovibrionales(100);Bdellovibrionaceae(100);Bdellovibrio(100); |
| 0139 | Bacteria(100);"Proteobacteria"(100);unclassified(100);unclassified(100);unclassified(100);unclassified(100); |
| 0142 | Bacteria(100);unclassified(100);unclassified(100);unclassified(100);unclassified(100);unclassified(100); |
| 0143 | Bacteria(100);"Proteobacteria"(100);unclassified(100);unclassified(100);unclassified(100);unclassified(100); |
| 0144 | Bacteria(100);OD1(100);OD1_class_incertae_sedis(100);OD1_order_incertae_sedis(100);OD1_family_incertae_sedis(100);OD1_genus_incertae_sedis(100); |
| 0145 | Bacteria(100);"Proteobacteria"(100);Alphaproteobacteria(100);Rhodospirillales(98);Rhodospirillaceae(97);unclassified(97); |
| 0146 | Bacteria(100);"Chlamydiae"(100);Chlamydiae(100);Chlamydiales(100);Parachlamydiaceae(100);unclassified(100); |
| 0148 | Bacteria(100);OD1(100);OD1_class_incertae_sedis(100);OD1_order_incertae_sedis(100);OD1_family_incertae_sedis(100);OD1_genus_incertae_sedis(100); |
| 0150 | Bacteria(100);OD1(93);OD1_class_incertae_sedis(93);OD1_order_incertae_sedis(93);OD1_family_incertae_sedis(93);OD1_genus_incertae_sedis(93); |
| 0155 | Bacteria(100);unclassified(100);unclassified(100);unclassified(100);unclassified(100);unclassified(100); |
| 0157 | Bacteria(100);unclassified(100);unclassified(100);unclassified(100);unclassified(100);unclassified(100); |
| 0159 | Bacteria(100);"Proteobacteria"(100);Gammaproteobacteria(100);unclassified(100);unclassified(100);unclassified(100); |
| 0163 | Bacteria(100);"Proteobacteria"(100);Deltaproteobacteria(100);unclassified(100);unclassified(100);unclassified(100); |
| 0164 | Bacteria(100);"Verrucomicrobia"(100);Opitutae(100);Opitutales(100);Opitutaceae(100);Opitutus(100); |
| 0169 | Bacteria(100);OD1(100);OD1_class_incertae_sedis(100);OD1_order_incertae_sedis(100);OD1_family_incertae_sedis(100);OD1_genus_incertae_sedis(100); |
| 0171 | Bacteria(100);"Proteobacteria"(100);Alphaproteobacteria(100);Rhizobiales(100);Xanthobacteraceae(91);Pseudolabrys(91); |
| 0180 | Bacteria(100);"Proteobacteria"(100);Deltaproteobacteria(100);Bdellovibrionales(100);Bdellovibrionaceae(100);Bdellovibrio(100); |
