# Supplementary material for: Spatial-Temporal Survey and Occupancy-Abundance Modeling To Predict Bacterial Community Dynamics in the Drinking Water Microbiome
Source: mBio. 2014 May 27;5(3):e01135-14. doi: 10.1128/mBio.01135-14 (PMC4045074; doi:10.1128/mBio.01135-14)
Supplement: Table S5 — Pipe characteristics for the three sectors of the Ann Arbor DWDS included in this study (Fig. 1). NA, not applicable. [file mbo003141850st5.docx]

**Supplementary Table 5**. Pipe characteristics for the three sectors of the Ann Arbor DWDS included in this study (Figure 1). N/A = not applicable.

| **Sector** | **Material** | **Pipe section** | **Pipe length**  **(km)** | **Surface area**  **(m^2^)** | **Pipe age**  **(years)** | |
| --- | --- | --- | --- | --- | --- | --- |
|  |  | **number** | **Cumulative** | **Cumulative** | **Average** | **Stdev** |
| S1 | Asbestos cement | 7 | 0.29 | 458.5 | 21 | 0 |
|  | Cast Iron | 71 | 4.12 | 6771.9 | 67 | 19 |
|  | Concrete | 8 | 2.58 | 6144.2 | 71 | 23 |
|  | Ductile Iron | 113 | 3.65 | 5441.2 | 20 | 20 |
|  | Polyvinyl chloride | 8 | 0.14 | 219.1 | 21 | 0 |
|  |  |  |  |  |  |  |
| S2 | Asbestos cement | N/A | N/A | N/A | N/A | N/A |
|  | Cast Iron | 90 | 5.86 | 9553.1 | 53 | 4 |
|  | Concrete | N/A | N/A | N/A | N/A | N/A |
|  | Ductile Iron | 153 | 6.95 | 11367.1 | 26 | 19 |
|  | Polyvinyl chloride | 7 | 0.02 | 14.6 | 6 | 0 |
|  |  |  |  |  |  |  |
| S2 | Asbestos cement | 3 | 0.07 | 115.8 | 48 | 12 |
|  | Cast Iron | 127 | 7.74 | 10849.6 | 55 | 7 |
|  | Concrete | N/A | N/A | N/A | N/A | N/A |
|  | Ductile Iron | 263 | 14.3 | 21151.2 | 32 | 16 |
|  | Polyvinyl chloride | 1 | 0.006 | 1.9 | 20 | N/A |
